# The rise and fall of countries in the global value chains

**DOI:** 10.1038/s41598-022-12067-x

**Published:** 2022-05-31

**Authors:** Luiz G. A. Alves, Giuseppe Mangioni, Francisco A. Rodrigues, Pietro Panzarasa, Yamir Moreno

**Affiliations:** 1 Department of Chemical and Biological Engineering, Northwestern University, Evanston, IL 60208 USA; 2 Department of Electrical, Electronic and Computer Engineering, University of Catania, 95125 Catania, Italy; 3 Institute of Mathematics and Computer Science, University of São Paulo, São Carlos, SP 13566-590 Brazil; 4 School of Business and Management, Queen Mary University of London, London, E1 4NS UK; 5 Institute for Biocomputation and Physics of Complex Systems (BIFI), University of Zaragoza, 50009 Zaragoza, Spain; 6 Department of Theoretical Physics, University of Zaragoza, 50009 Zaragoza, Spain; 7 ISI Foundation, 10126 Turin, Italy

**Keywords:** Complex networks, Applied mathematics, Statistical physics

## Abstract

Countries become global leaders by controlling international and domestic transactions connecting geographically dispersed production stages. We model global trade as a multi-layer network and study its power structure by investigating the tendency of eigenvector centrality to concentrate on a small fraction of countries, a phenomenon called localization transition. We show that the market underwent a significant drop in power concentration precisely in 2007 just before the global financial crisis. That year marked an inflection point at which new winners and losers emerged and a remarkable reversal of leading role took place between the two major economies, the US and China. We uncover the hierarchical structure of global trade and the contribution of individual industries to variations in countries’ economic dominance. We also examine the crucial role that domestic trade played in leading China to overtake the US as the world’s dominant trading nation. There is an important lesson that countries can draw on how to turn early signals of upcoming downturns into opportunities for growth. Our study shows that, despite the hardships they inflict, shocks to the economy can also be seen as strategic windows countries can seize to become leading nations and leapfrog other economies in a changing geopolitical landscape.

## Introduction

One of the defining features of modern production systems is the organization of value chains into distinct stages that are geographically spread out across the entire globe and to which countries contribute in complex and non-linear ways. Spatially disaggregated production systems result in a worldwide trade network in which companies of multiple countries from different production sectors exchange intermediary products along a multi-stage, non-linear, and geographically boundless production trajectory ending with products and services directed at the final demand^1^. In this network, strategic access to scarce resources plays a critical role in shaping international relationships between countries and across industries, and in enabling countries to secure and maintain economic prominence globally and over time^2^. The study of how products and services flow within countries and from exporters to importers is therefore crucial to better understand how countries can secure prominent roles in the global value chains.

Recent years have witnessed a growing number of network studies concerned with the worldwide trade system^3–13^. Using simplex networks to represent countries as nodes and transactions as directed links from exporters to importers, it has been suggested that the worldwide trade network exhibits a community structure^14,15^, a heavy-tailed degree distribution^6^, and small-world properties^13^. By extending the simplex representation to bipartite networks, in which nodes in one class can only connect to nodes in another class^16^, researchers have focused on early signals of economic downturns^17^, the competitiveness of countries^18^, and the complexity of products^11^.

In spite of these recent advances^19^, a number of scholars have highlighted the limitations of simplex network representations and projected networks, and in particular have emphasized how these network paradigms can poorly capture the dynamics of complex systems^20,21^. In contexts where the system has multiple layers interconnected with each other—such as, for example, the case of transportation systems where fluxes of individuals traveling by bus, train, and airplane can be seen as belonging to different layers of the same interconnected network—the multi-layer network has been shown to provide a more adequate representation^21–23^. This is also the case of the international trade network, in which the structure unfolds within and across industries, and the countries are involved in multiple stages of production along the global value chains^24^. In the multi-layer representation, industries refer to the layers of the network, the countries are the nodes that populate every layer, and connections can be drawn from one country to another in the same industry (within-layer connections) or between different industries (cross-layer connections)^24,25^.

Over recent years, researchers have adopted a multi-layer perspective to better capture the structure and dynamics of international trade^7,24–28^. Applications of multi-layer networks to trade include the analysis of layer-specific local constraints on international trade^26^, the study of the emergence and unfolding of cascading failures^27^, the study of countries’ influence based on betweenness centrality measures^28^, the analysis of the nested structural organization of worldwide trade^25^, and the assessment of the impact of technological innovations on industrial products^7^, to name only a few. However, despite the increasing popularity of network approaches to global trade, relatively little attention has been paid to the formalization of countries’ economic dominance from a multi-layer perspective. Traditionally, scholars and policy-makers interested in comparative assessments of countries’ global roles and competitive advantage have relied on macro-economic measures of market power based on countries’ overall share of world trade^29^. These traditional aggregate measures, however, suffer from shortcomings, mainly because aggregate trade flows, on which dominance is predicated, cannot account for the increasingly widespread internationalization of production processes^4,30,31^.

Countries’ economic dominance of global trade is intrinsically rooted in the international fragmentation of production and the resulting structural intricacies that characterize the global value chains. Production processes are typically disaggregated into various stages stretching across multiple countries so as to exploit the comparative advantages of locations. For example, production might involve intermediary stages and assembly spots located in countries that hold only a negligible share of the market of the final product. Countries may receive inputs from multiple suppliers and, in turn, contribute to the production process at multiple stages located in various other countries. Thus, the internationalization of production makes it difficult to assess countries’ role in global trade simply by using traditional aggregate values such as gross imports or exports. By contrast, a country’s economic dominance of global trade should be a function of the share of value the country brings to each production stage of the underlying global value chains and of the share of benefits obtained from the exchange of intermediate and final products^32,33^. That is, economic dominance is a multi-layer property that should quantify the salience of a country for the entire production network, and thus explicitly depend on the centrality of the other countries located at other production stages and with which the focal country is connected.

Here, we take a step in this direction, and investigate the dynamics of dominance in the worldwide multi-layer trade network from 2000 to 2014. First, we take a micro perspective and investigate the dynamics of the economic dominance of individual buyers and sellers in global trade. We show that the ranking of buyers and sellers varied over time in a non-trivial way, resulting in an abrupt and remarkable reversal of leading roles precisely before the 2008 financial crisis. Using hierarchical clustering analysis, we uncover common patterns of variation in economic dominance, and use these patterns to partition countries into meaningful groups characterized by similar power dynamics. We then shift focus from individual countries and take a macro perspective. First, we identify a localization effect in the system using the inverse participation ratio, and examine whether variations in localization can be associated with the occurrence of exogenous shocks. Second, we shed light on the contributions of individual industries to countries’ global dominance, and uncover the localization effect within each industry by computing the corresponding inverse participation ratio. Finally, we explore the role that domestic trade played in amplifying the system’s power concentration and facilitating variations in the geopolitical landscape before the 2008 crisis.

## Results

### The multi-layer network

**Figure 1:**
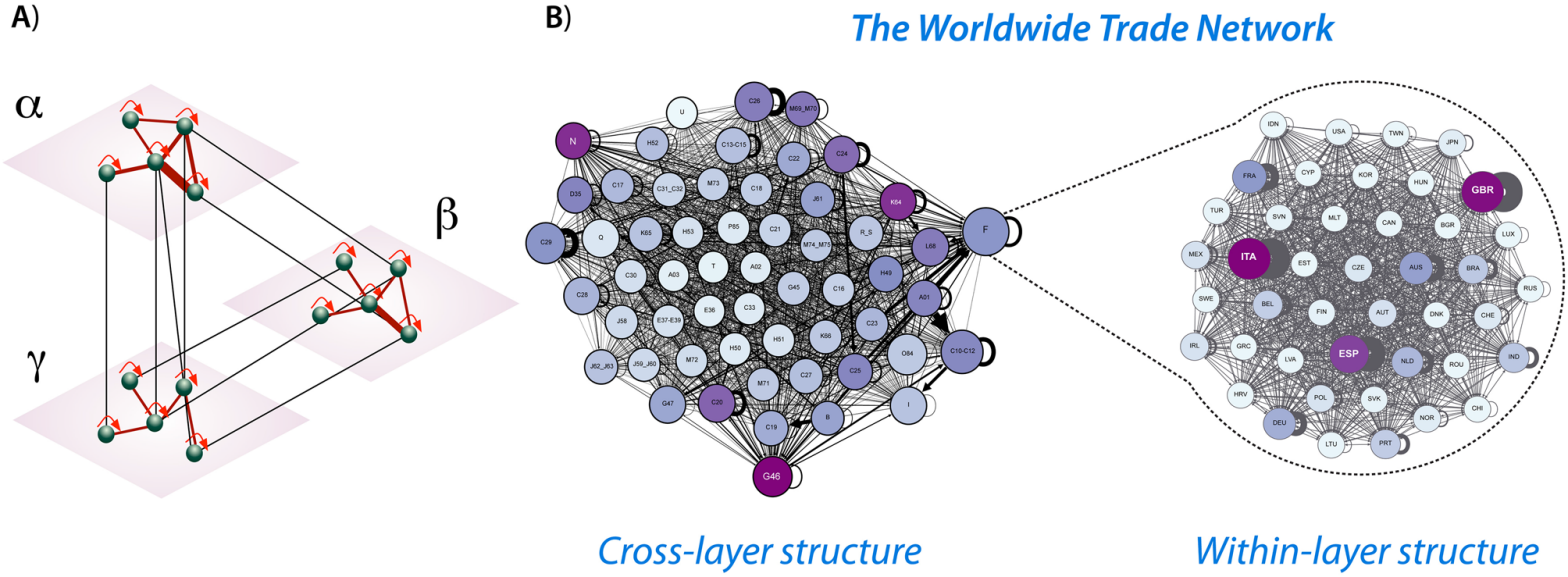
The worldwide trade multi-layer network. (**A**) Schematic representation of the multi-layer network. Each node represents a country and each layer an economic industry. The edges represent economic transactions starting from the sellers and pointing to the buyers. The widths of edges are proportional to the USD value of the goods or services exchanged between the connected countries. (**B**) The worldwide trade network in 2000. For illustrative purposes, we show: (i) the aggregated cross-layer structure of the network, where edges refer to connections among countries from different industries; and (ii) an example of within-layer structure, where edges refer to connections between countries within the construction industry. The size of each node is proportional to the in-strength of the node, $s^{in}$, whereas the color intensity of each node is proportional to the out-strength of the node, $s^{out}$. See Supplementary Tables I and II for the labels of the nodes.

Our analysis begins with the construction of the multi-layer network using data from the World Input-Output Database (WIOD)^34^ (Fig. 1). The WIOD is an information-rich data set including details about trade among 56 industries and 43 countries accounting for more than 85% of the overall global GDP in the period from 2000 to 2014. Unlike other data sets (e.g., COMTRADE), the WIOD has information about connections between different industries and different countries. Thus, despite the limited time interval of 14 years, the WIOD data set would enable us to examine the dynamics of the various intermediate production stages in the global value chains. To this end, we construct a weighted directed multi-layer network, where each industry is represented as a layer and every layer is populated by the 43 countries of the data set, which are connected when they trade with one another^24,25^. In particular, within-layer connections refer to the exchange of products and services among countries within the same industry, whereas cross-layer connections refer to economic transactions among countries in different industries (see Fig 1 and “Methods”).

### Economic dominance of buyers and sellers

In graph theory, a common measure for assessing the importance of nodes in a network is the eigenvector centrality^16,35,36^. The idea underpinning this measure is that a node is important to the extent that it is connected to other important nodes, and thus belongs to a chain in which importance is transmitted from one node to another along various connections^35^. Research has shown that under commonly occurring conditions (e.g., in networks with heavy-tail distributions) the eigenvector centrality undergoes a localization transition as a result of which most of the weight of the centrality will concentrate on a small number of nodes in the network, while the vast majority of the remaining nodes will be assigned only a vanishing fraction^37–39^. This phenomenon, typically referred to as “localization effect”, has been extensively studied in the case of simplex complex networks^40^, and more recently it has been investigated in multi-layer networks^37–39^. Traditionally the literature has regarded the localization effect as a drawback of the eigenvector centrality, which in turn has motivated the proposal of alternative centrality measures better able to assess the relative importance of peripheral nodes that would otherwise remain indistinguishable. Here, we take a different perspective: we shift focus from the ranking of peripheral nodes to the emergence of power structures in which a minority of nodes take on a leading role. In particular, we explore how the localization process in the multi-layer trade network can unmask important structural changes in global trade associated with the emergence of a few dominant players. We also assess how these changes can result in transformations of power hierarchies in the global value chains, and ultimately affect the relative economic dominance of the leading global suppliers and destination markets.

First, we define the economic dominance of a country from a network-based perspective. More generally, a country can be seen as a global leader when it exerts control over the whole range of transactions occurring along the international global value chains. This implies that a country’s global dominance should be a function of the dominance of all its trading partners at the local levels of the upstream, midstream, and downstream stages of production within and across all industries. Moreover, countries can be global leaders as both buyers and sellers. On the one hand, a country is a leading global buyer to the extent that it represents a major destination market of intermediary and finished products sold by countries that, in turn, are key destination markets of products originating from other key destination markets, and so forth. On the other, a country is a leading global supplier to the extent that it controls the sales of intermediary and finished products to countries that, in turn, are key suppliers of products to other key suppliers, and so forth.

We measure the economic dominance of buyers and sellers using a suitable adaptation of eigenvector centrality to the multi-layer network. The centrality of nodes can be measured through the tensorial formulation of the multi-layer network by calculating the spectral properties of the graph. Specifically, the corresponding formalization of the multi-layer network in the tensorial notation is rank-4 tensor $M_{i\alpha }^{j\beta }$, which encodes a directed, weighted connection between node *i* from layer $\alpha$ to any other node *j* in any layer $\beta$^38,39,41^. Thus, we can compute the centrality of buying nodes in a given layer by calculating the leading eigentensor associated with the most positive eigenvalue^38^. To also account for sellers, we can use the left leading eigentensor when calculating the centrality (or, equivalently, we can calculate the leading eigentensor associated with the most positive eigenvalue of the transposed supra-matrix). Thus, the node centrality can be obtained by solving the following equation^41^:

1 $$\begin{aligned} M_{i\alpha }^{j\beta }\Theta _{i\alpha }=\lambda \Theta _{j\beta }, \end{aligned}$$

where $\Theta _{i\alpha }$ gives the eigenvector centrality of each node *i* in each layer $\alpha$ when accounting for the whole interconnected multi-layer structure. The centrality of each node in the whole multi-layer network is given by aggregating over the layers the centrality of each node in each layer. Formally, this is equivalent to multiplying $\Theta _{i\alpha }$ by a rank-1 tensor ($u^\alpha$) with all components equal to 1^37,38^, namely

2 $$\begin{aligned} \theta _i^M=\Theta _{i\alpha }u^\alpha . \end{aligned}$$

**Figure 2:**
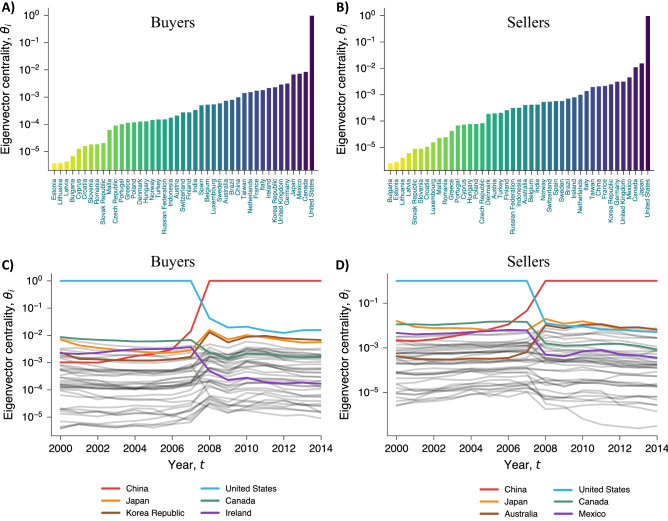
Dynamics of economic dominance. The eigenvector centrality of buyers and sellers in the worldwide trade multi-layer network in 2000 is shown in panels (**A**) and (**B**), respectively. Countries are ordered by their centrality from the least central to the most central. The rankings of buyers and sellers show some similarity, as suggested by the Kendall-$\tau$ correlation coefficient (0.82 with a *p* value of $<10^{-14}$). Countries that are central buyers tend to be also central sellers. The variation of eigenvector centrality over time for buyers (**C**) and sellers (**D**) shows the emergence of new winners and losers just before the 2008 financial crisis. The centralities of China and the US are shown in red and blue solid lines, respectively. Panels (**C**) and (**D**) highlight the three countries that experienced the largest positive and negative variations in eigenvector centrality between 2007 and 2008, respectively in the buyers’ and sellers’ markets.

Solving the equation above for buyers and sellers in the multi-layer network in the year 2000, the eigenvector centrality ranks the US as the dominant country both as a buyer and as a seller. The least dominant countries in 2000 are Estonia and Bulgaria, appearing at the bottom of the rankings of buyers and sellers, respectively. The values of eigenvector centrality in the multi-layer network for this year are shown in Fig. 2A (buyers) and Fig. 2B (sellers). The rankings of buyers and sellers show some similarity, as indicated by the Kendall-$\tau$ correlation coefficient (0.82; *p* value $<10^{-14}$). Countries that are prominent destination markets tend to be also prominent suppliers. The majority of countries change ranks only by a position or two, with the noticeable exception of Luxembourg, which occupies position number 17 in the ranking of buyers and position number 36 in the ranking of sellers.

We now focus on the dynamics of dominance in the worldwide trade multi-layer network. We computed the eigenvector centrality for each year in the period from 2000 to 2014, for both buyers (Fig. 2C) and sellers (Fig. 2D). Interestingly, our results suggest that an abrupt reversal of economic dominance between the US and China took place precisely just before the 2008 global financial crisis. Findings also clearly indicate that many other countries changed their global roles during the crisis, and new winners and losers emerged in a reshaped geopolitical landscape. In particular, in addition to the US and China, Fig. 2C,D highlight, respectively, the three buyers and the three suppliers that experienced the largest positive and negative variations in eigenvector centrality between 2007 and 2008. For example, in 2008, like China, Japan emerged as a prominent economy in global production, whereas Canada followed the same trajectory as the US, permanently losing the role occupied before the crisis as the world’s second most prominent trading nation.

### Blocs of economic dominance

**Figure 3:**
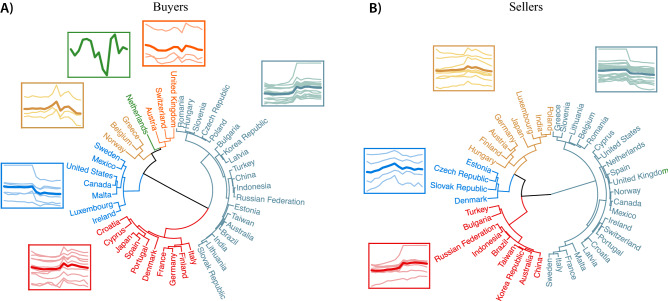
Hierarchical structure of the worldwide trade network. The dendrograms show the result of the hierarchical clustering based on Pearson’s correlation distance using the Ward linkage criteria for buyers (**A**) and sellers (**B**). The colors indicate the clusters obtained by cutting the dendrograms at the threshold distance that maximizes the silhouette score. The insets show the time series of the eigenvector centralities (thinner lines in light-shaded colors) of the countries belonging to each cluster (y-axis on log-scale). The thicker lines refer to the average trends of each cluster. The buyers’ hierarchical structure has six clusters, whereas the sellers’ hierarchical structure has only four.

To further understand the emergence and evolution of groups of trading countries, we investigate the hierarchical structure of countries based on dynamics of economic dominance. By computing the Pearson’s correlation distance matrix (see “Methods” and Supplementary Fig. 1) and using the Ward linkage criteria^42,43^, we constructed a dendrogram that hierarchically clusters similar time series of eigenvector centrality. This clustering procedure recursively merges pairs of clusters that minimally increase within-cluster variance. We determined the number of significant clusters by cutting the dendrogram at the threshold distance that maximizes the silhouette score^44,45^. Figure 3 shows the obtained clusters of buyers (A) and sellers (B) and the corresponding average trends of eigenvector centrality (see also the matrix plot of the correlation distance among all pairs of time series in Supplementary Fig. 1). To check for robustness, we also computed the modular structure of the network using a hierarchical (nested) stochastic block model, and obtained a substantial overlap with the partitioning based on the hierarchical cluster analysis (see Supplementary Fig. 2).

Findings suggest that destination markets and suppliers can be grouped according to similar patterns of variation in the role they occupied in the economic system over time. In particular, Fig. 3A shows that buyers are grouped into six economic blocs, the largest of which (by number of members) contains 19 countries (including Brazil, Russia, India, China). This bloc accounted for $20\%$ of all purchases in 2000 and overtook all the other blocs by 2007, reaching $\approx 50\%$ of all purchases in 2014 (see Supplementary Fig. 3A). All the other blocs reduced their share of global purchases over time. The second-largest bloc includes 10 countries (9 European countries and Japan), and accounted for $20\%$ of all purchases in 2014. The third bloc, including 8 countries (five European countries and the three North American countries, namely, Canada, the US, and Mexico), boasted the largest share of all purchases in 2000 (approximately $36\%$), but played a weaker role in 2014 ($24\%$ of global purchases). The other two clusters include three countries each (all from Europe, representing about $5\%$ of all trades, in 2014); the remaining cluster is composed of a single buyer (the Netherlands), and accounts for about $1\%$ of all purchases during the period.

Like buyers, sellers can be hierarchically grouped into clusters characterized by similar variations of economic dominance. In particular, as shown by Fig. 3B, sellers are partitioned into four economic blocs, the largest of which includes 22 countries (19 from Europe and the other three from North America, namely, Canada, the US, and Mexico). This bloc accounted for $56\%$ and $40\%$ of all global sales, respectively in 2000 and 2014 (see Supplementary Fig. 3C). The second-largest economic bloc, which includes 9 countries (six Asian countries, Australia, Brazil, and Bulgaria), overtook the largest bloc only in 2013, with $43\%$ of all sales in 2014. The third economic bloc includes 8 countries (six European countries and two Asian countries) and accounted for less than $2\%$ of all sales in 2014. Although the last economic bloc is the smallest by number of countries (only four European countries), it accounted for $15\%$ of all sales in 2014.

We now shed some light on the drivers of such partitioning. First, it is interesting to examine the extent to which the grouping of countries based on patterns of variation in economic role overlaps with a more traditional partitioning based on the idea that countries are more likely to trade within than between groups^46^. While Supplementary Fig. 3B,D seems to suggest that over the years the total purchase taking place within blocs represents a fraction that varies from 65% (for the smallest blocs) to 95% (for the largest blocs), this is only true when domestic trade is accounted for. Indeed, as shown in Supplementary Fig. 4, when the analysis is restricted only to international trade, the reverse is true. Except for the third-largest bloc of importers (including the US, Canada, and Mexico) and the largest bloc of exporters (still including the three North American countries), countries tend to trade between rather than within blocs. Thus, the majority of trade occurring within blocs tends to originate from domestic trade, i.e., self-loops within and across layers (see Supplementary Fig. 5). As suggested by Supplementary Figs. 6–11, the economic value of the transactions within blocs does not statistically significantly differ from what would be expected by chance (at the 0.05 significance level), except for the third-largest bloc of importers (including the US, Canada, and Mexico) and the largest bloc of exporters (still including the three North American countries). Countries with similar power dynamics tend, therefore, to avoid trading with each other, and instead concentrate their transactions with partners belonging to other clusters.

Second, the obtained groups have a geographical signature too. As suggested by Supplementary Tables III and IV, the mean geographic distance between countries within the same bloc (computed using the geographical centroids of countries) is always lower (except for the largest bloc of buyers) than the mean distance separating countries from different blocs. Thus, spatial proximity can be regarded as a fundamental driving force underpinning the formation of clusters of trading countries characterized by comparable dynamics of economic dominance (see also the geographic mapping of countries in Supplementary Fig. 12).

### Inverse participation ratio

**Figure 4:**
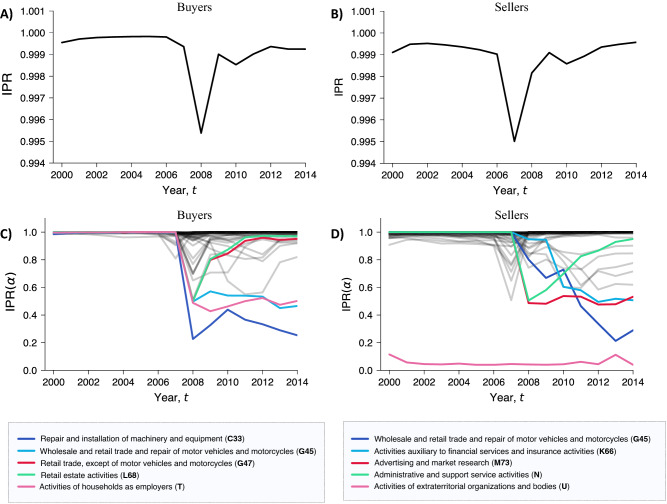
Dynamics of the inverse participation ratio (IPR). (**A-B**) The panels show a drop in the IPR during the financial crisis. This happened between 2007 and 2008 for the buyers (**A**) and between 2006 and 2007 for the sellers (**B**). (**C–D)** Contribution of industries to buyers’ and sellers’ dominance. Contributions of an individual layer $\alpha$ to the inverse participation ratio (IPR$(\alpha )$). The colored industries are the ones that experienced the largest drop in IPR$(\alpha )$ during the observed period. Notice that most of the industries that experienced a drop in localization did not revert back to the initial localized state.

To further investigate the dynamics of eigenvector centrality, we computed the inverse participation ratio (IPR) of buyers and sellers. From network theory^39,47^, we know that

3 $$\begin{aligned} \text {IPR}=\sum _i \theta _i^4, \end{aligned}$$

where $\theta _i$ is the eigenvector centrality of node *i*. An IPR close to zero means that there is negligible localization effect (i.e., no economic dominance), and centrality is homogeneously distributed across the nodes, whereas an IPR close to one reflects a network where the centrality is very localized in very few nodes (i.e., the system is dominated by a minority of countries)^47^.

Using Eq. (3), we computed the IPR for the worldwide trade multi-layer network over the period from 2000 to 2014, for buyers (Fig. 4A) and sellers (Fig. 4B). Findings clearly suggest a drop in the localization effect that took place precisely before the 2008 financial crisis. Combined with the observed changes in countries’ eigenvector centrality (see Fig. 2), the drop in localization indicates a more homogeneous redistribution of economic dominance across trading countries. Notice that, despite the similar shape, the drop in IPR for sellers precedes in time the one for buyers. This may suggest some linkages between global supply and destination markets: early-warning structural signals of the 2008 crisis started to appear in the sellers’ market and then propagated along the global value chains to also affect the structure of the global purchasing market.

To assess the statistical significance of the high values of IPR (i.e., to test the hypothesis that the observed values of IPR values could be generated by chance), we used a random-edge assignment procedure to construct an ensemble of synthetic multi-layer networks, with 1000 realizations, and with the same topological features as the empirical networks. To produce the null model, we randomly redirected the in- or out-going edges by preserving their weights, thus also preserving the in- or out-degree distributions and the in- or out-strength distributions in the different layers, and then computed the eigenvector centrality (see “Methods”). So constructed, this null model preserves the in- and out-degree distributions, in-, and out-strength distributions, as well as the weight distribution at three levels: (1) globally, in the entire network; (2) at the level of cross-layer connections; and (3) locally within each layer. For each synthetic multi-layer network, we computed the IPR values for buyers and sellers to obtain a distribution of values that can then be compared with the results obtained from the empirical multi-layer networks. Assuming a 5% false discovery rate, we can reject the hypothesis that the localization effect found in the multi-layer network can be generated by chance using a null model that has the same topological features as the real multi-layer network (see Supplementary Fig. 13).

**Figure 5:**
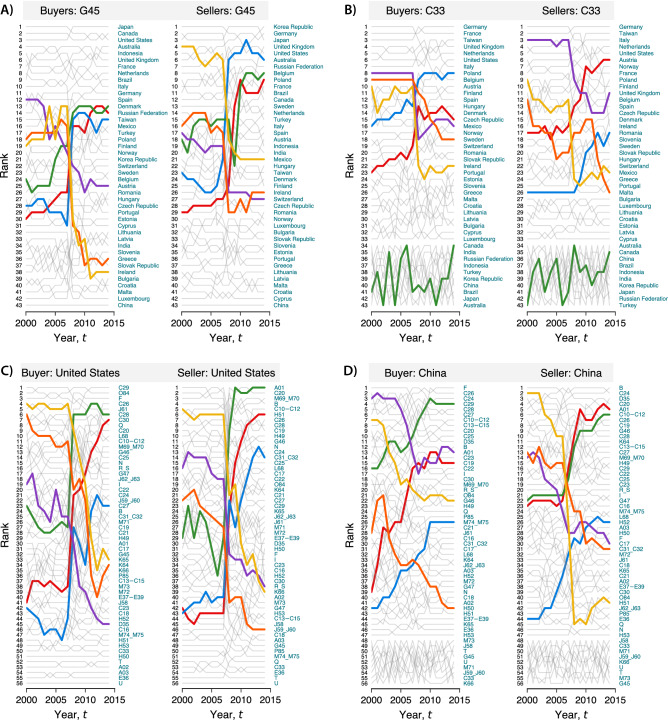
Variation in eigenvector centrality of countries within individual industries and of industries within countries. In each panel, the left-hand *y*-axis shows the rankings of countries (**A,B**) or industries (**C,D**) in 2000, and the right-hand *y*-axis shows the same rankings in 2015. Each panel highlights the top three countries (**A,B**) or industries (**C,D**) with the largest increase and decrease in ranking over the whole period. (**A**) Winners and losers in the industry of wholesale and retail trade and repair of motor vehicles and motorcycles (G45). (**B**) Winners and losers in the industry of repair and installation of machinery and equipment (C33). (**C**) Global purchases and sales of the US. The top three purchasing industries with the largest increase in ranking were manufacture of other transport equipment (C30—red), manufacture of electrical equipment (C27—blue), and manufacture of machinery and equipment n.e.c. (C28—green), whereas the purchasing industries with the largest decrease in ranking were electricity, gas, steam, and air conditioning supply (D35—purple), financial service activities, except insurance and pension funding (K64—orange), and insurance, reinsurance, and pension funding, except compulsory social security (K65—yellow). The top three supplying industries with the largest increase in ranking were air transport (H51—red), manufacture of furniture, other manufacturing (C31–C32—blue), and crop and animal production, hunting, and related service activities (A01—green), whereas the three supplying industries with the largest decrease in ranking were “other” service activities (R-S—purple), motion picture, video and television program production, sound recording and music publishing activities, programming and broadcasting activities (J59–J60—orange), and activities auxiliary to financial services and insurance activities (K66—yellow). (**D**) Global purchases and sales of China. The top three purchasing industries with the largest increase in ranking were manufacture of coke and refined petroleum products (C19—red), other professional, scientific, and technical activities, and veterinary activities (M74–M75—blue), and manufacture of motor vehicles, trailers, and semi-trailers (C29—green), whereas the three purchasing industries with the largest decrease in ranking were crop and animal production, hunting and related service activities (A01—purple), water transport (H50—orange), wholesale trade, except motor vehicles and motorcycles (G46—yellow). The top three supplying industries with the largest increase in ranking were crop and animal production, hunting and related service activities (A01—red), warehousing and support activities for transportation (H52—blue), other professional, scientific, and technical activities, and veterinary activities (M74–M75—green), whereas the three supplying industries with the largest decrease in ranking were manufacture of paper and paper products (C17—purple), manufacture of furniture and other manufacturing (C31–C32—orange), and air transport (H51 0 yellow). The full list of labels for industries can be found in Supplementary Table II.

We investigate the contribution of individual industries to the localization effect observed globally in the system^39^. To this end, we computed the contribution of individual layers to the IPR using the following measure:

4 $$\begin{aligned} \text {IPR}(\alpha )=\sum _k (\theta _k^{M[\alpha ]})^4, \end{aligned}$$

where $\theta _k^{M[\alpha ]}$ is the eigenvector centrality of node *k* in layer $\alpha$. Using this measure, we can uncover the industries that most contributed to the global localization effect, from the perspective of both buyers and sellers. Figure 4C,D shows the curves obtained for purchases and sales, respectively, in each layer $\alpha$ and highlight the industries characterized by a major drop in IPR in the period. The figure suggests that, for both purchases and supplies, the values of IPR($\alpha$) dropped just before the 2008 crisis. While many of the industries returned to a highly localized state (IPR $\approx 1$), other industries remained less localized and more homogeneous by distribution of power. For example, purchases in retail trade (not including motor vehicles and motorcycles) and real estate activities experienced a decay of localization during the crisis but quickly returned to a centralized power structure. By contrast, purchases within the industries of repair and installation of machinery and equipment, wholesale and retail trade and repair of motor vehicles and motorcycles, and household activities lost their market power concentration in 2007. Similar patterns can be found with suppliers. For example, administrative and support service activities experienced a decay in localization, but then slowly returned to a more centralized structure. On the other hand, other industries such as the wholesale and retail trade and repair of motor vehicles and motorcycles, activities auxiliary to financial services and insurance activities, and advertising and market research remained less centralized for the rest of the period.

We now illustrate how countries experienced variations in economic dominance within individual industries and how individual industries contributed to the rise and fall of countries. To this end, here we focus on the two industries that experienced the largest variation in the power concentration of purchases and supplies (i.e., repair and installation of machinery and equipment, and wholesale and retail trade and repair of motor vehicles and motorcycles, respectively; see Fig. 4C,D) and the two countries with the largest variation in economic dominance (i.e., the US and China; see Fig. 2C,D).

To calculate the ranking of countries within individual industries, we computed the eigenvector centrality $\theta _{i \alpha }$ for each buyer (seller) *i* in each industry $\alpha$ given by the eigenvector centralities of the supra-adjacency matrix (Eq. 2). We then computed the ranking of countries, for a given industry $\alpha =\alpha ^*$, by sorting $\theta _{i \alpha ^*}$ from the highest to the lowest value. Similarly, we computed the ranking of industries $\alpha$, for a giving country $i=i^*$, by sorting $\theta _{i^* \alpha }$ from the highest to the lowest value. The above procedure is then repeated for every year in our data set. Results for the two industries and the two countries are shown in Fig. 5. It is worth noting that the countries with a dominant position in these industries (see the five top-ranked countries in Fig. 5A,B) or the countries with a negligible role (see the five lowest-ranked ones in Fig. 5A,B) tend to maintain their positions over time. The “market movers” are the countries that typically occupy the middle of the ranking. Figure 5C,D sheds more light on how industries contributed to the power dynamics of the US and China and to the reversal of leading role between the two countries between 2007 and 2008. For example, the US experienced its largest loss of market dominance in the purchase of electricity, gas, steam, financial services, and insurance, and in the supply of other service activities, television program production, broadcasting, music publishing, and broadcasting activities. By contrast, China became a global leader by strengthening its market position in the purchase of coke, refined petroleum products, scientific and technical activities, and motor vehicles, and in the supply of crop and animal production, warehousing, and support activities for transportation. Once again, most of these gains and losses in competitive advantage took place in 2007 before the crisis.

### The role of domestic trade in localization transition

So far our study has suggested that localization varies across industries, and some industries are more localized than others. To further understand the sources of localization and the reversal of dominance between countries, we explore the distinct contribution to the localization of international trade (i.e., the edges connecting different countries within the same layer or across different layers) and domestic trade (i.e., self-loops within layers and cross-layer connections involving the same country). To this end, we first disaggregated our supra-matrix $\mathbf{M}$ into an international trade matrix $\mathbf{T}$ and a domestic trade matrix $\mathbf{D}$. The domestic trade matrix includes all diagonals of each layer (i.e., the self-loops connecting a country with itself in the same layer) as well as the diagonals of the non-diagonal matrices that represent the cross-layer connections of a country with itself in different layers. We then simulated different scenarios of trade by multiplying $\mathbf{D}$ by a parameter *c*. This parameter *c*, therefore, accounts for the role played by domestic transactions in the network. Thus, *c* varies from 0, when all the domestic trade is removed from the network, to 1, where the original topology of the multi-layer network remains unchanged. We can formally define a new multi-layer network as the sum of international trade and domestic trade:

5 $$\begin{aligned} {\tilde{\mathbf{M}}}=\mathbf{T}+ c\, \mathbf{D}, \end{aligned}$$

where *c* is our control parameter.

**Figure 6:**
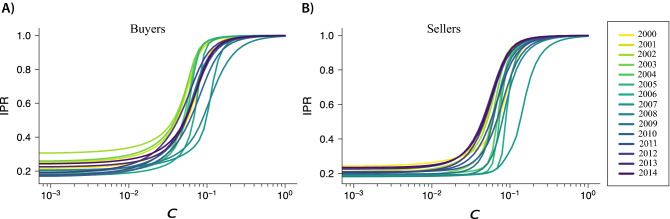
The transition to a localized state in the multi-layer network depends on domestic trade. By decomposing the supra-matrix into the sum of international trade and domestic trade, we use *c* as a control parameter for varying the percentage of domestic trade accounted for when computing the IPR (Eq. 5). Each line shows the values of IPR calculated in a specific year and for different values of *c*. Findings suggest a transition to the localized state when $c\approx 0.08$. The panels show the localization transition for (**A**) buyers and (**B**) sellers in each year from 2000 (yellow) to 2014 (purple).

Next, for each year in the data set, we calculated the IPR of ${\tilde{\mathbf{M}}}$ by using different values of *c* and, as usual, by distinguishing between the eigenvector centralities of buyers and sellers. Figure 6 shows the IPR as a function of *c*. Results clearly indicate an abrupt transition to the localized regime at $c\approx 0.08$, thus suggesting that domestic trade was the main driver of localization in the network. The figure also suggests that the salience of domestic trade for localization is time-dependent. Interestingly, Supplementary Fig. 14 shows that the year-dependent critical value of the parameter *c*, at which the largest variation in IPR occurs, peaks precisely just before the financial crisis, both for buyers (see Supplementary Fig. 14A) and sellers (see Supplementary Fig. 14B). That is, at the time preceding an exogenous shock it takes a higher share of domestic trade to make the global market more heterogeneous and dominated by a minority of countries. This implies that, from the perspective of a given individual country, leveraging domestic trade to increase or cement the country’s market dominance becomes even more critical during a crisis.

Indeed the role played globally by domestic trade in boosting localization is reflected locally at the country’s level. As shown by Supplementary Fig. 15C, unlike the US and other countries, China was the only major economy to witness an uninterrupted surge in domestic trade during and beyond the financial crisis. On the other hand, the way China’s international trade varied during the crisis did not differ much from what occurred in the US and Germany (see Supplementary Fig. 15A,B). It is, therefore, the sustained and uninterrupted growth in domestic trade that enabled China to bolster its dominance of global trade, and eventually to overtake the US as the leading economy in the global value chains.

## Discussion

Countries’ rising market power and resilience against external shocks have become a prominent public policy issue in recent years. Here we have investigated dynamics of economic dominance using countries’ eigenvector centrality in the trade multi-layer network. Our findings have uncovered a localization effect pointing to a concentration of market power on a select minority of buyers and sellers. By examining the evolution of countries’ economic dominance, we concentrated on two main findings. First, a reverse of market dominance between the two major economies—the US and China—took place precisely before the 2008 financial crisis. At the same time, other countries abruptly changed their global roles, and new winners and losers emerged. Second, at the global level, a drop in localization took place before the crisis. In particular, the sellers’ global market was the first to exhibit a significant change in power structure in 2007. These changes subsequently reverberated through the network to also affect the buyers’ market.

By comparing the time series of countries’ market dominance over time, we identified a number of common patterns according to which we clustered the countries. We found that similarity in power dynamics does not serve as a catalyst for trade. In fact, trade tends to concentrate between countries that are geographically close, yet characterized by different trends of economic dominance. Moreover, our multi-layer framework enabled us to identify the distinct contributions of individual industries to localization. While most industries managed to react to the financial crisis by retaining a heterogeneous power structure, some experienced a drop in concentration without being able to revert back to the structure they held before the crisis. This is the case, for example, of the industries of repair and installation of machinery and equipment and wholesale and retail trade and repair of motor vehicles and motorcycles.

While policy-makers often focus on the role of exports in boosting a country’s economic power in global trade, the role of domestic trade has remained largely overlooked. Our analysis of the relationship between domestic trade and localization uncovered two main findings. First, domestic trade was the main driver of heterogeneity in the global power structure. Second, the salience of domestic trade for localization followed a time-dependent pattern: that is, domestic trade became most crucial for inducing power concentration in the system precisely just before and during an economic downturn. Whilst in normal times exports and, more generally, international trade enabled the US to cement its leading role, during the 2008 crisis it turned out to be domestic trade within and across industries that enabled China to overtake the US as the global market leader.

Overall, these findings have implications that straddle research and practice. Existing economic theories make divergent predictions on how the emergence of new dominant players impacts on the global political and economic landscape^48^. There is little consensus on whether an increased unbalance in market power is likely to affect economic dynamism, income inequality, and geopolitical stability^49^. The lack of consensus partly reflects the difficulties in measuring economic power, both at the level of a firm and of a country. For example, previous macroeconomic studies have traditionally used aggregate measures such as total exports or imports to gauge the role of countries in global trade^4^. However, recent studies have pointed to the inadequacy of such measures to reflect the intricacies of the underlying global value chains^30,31^. Here we did not perform a comparative assessment of alternative measures, but focused only on eigenvector centrality applied to the multi-layer network. Interestingly, the localization transition that the network literature has highlighted as a potential drawback of eigenvector centrality turned out to unmask properties of the power structure that would otherwise have remained hidden with other approaches. For example, our study helps to better understand how a global crisis can affect the much-debated trade war between the US and China and shift the balance of power between the two nations. The reversal in leading role that happened in 2007 suggests that exogenous events, like a financial crisis, can be turned into opportunities for global leadership. Eventually, it will be the nation that most effectively responds to such events that will gain traction and emerge as a global leader.

Recently there have been a number of attempts in the economic literature to cluster countries into meaningful blocs with distinctive preferential trade patterns within specific industries^14,15^. Here we took a different perspective on partitioning. We clustered countries according to the similarity of their time series of eigenvector centrality in the global value chains, and then evaluated the obtained clusters by inferring preferential trade patterns. Our approach allowed us to complement more traditional topological approaches as we uncovered associations between countries’ market positioning and preferential trading. Our partitioning suggests that countries with similar power dynamics (and thus belonging to the same bloc) tend to avoid trading with one another and concentrate transactions with other partners in different power-based blocs.

Our study contributes to the emerging literature on the structural early signals of economic downturns^17^, and more generally on the topological antecedents and consequences of shocks in economic and financial systems^50^. We did not assess any causal relation between structural changes and exogenous events, but simply the association between the timing of a financial shock and the emergence of new winners and losers in the market. All the analysis suggests is that changes in localization can be seen as a topological signal of an upcoming global crisis and at the same time as an opportunity for countries to develop effective strategies for safeguarding and strengthening their market positions. China serves as a case in point. The onset of the financial crisis in 2008 spurred an abrupt reversal of roles leading China to cement its status as the world’s dominant trading nation underpinned by an uninterrupted surge in domestic trade. While the role of countries in global trade has traditionally been gauged mainly based on exports and more generally international trade, this finding may suggest a change in perspective. First, an assessment of countries’ market dominance needs to take into account not only the role of exports and imports but also of domestic trade taking place within the global value chains. Second, the role that domestic trade played in the geopolitical landscape in 2007 has the potential to assist governments, world leaders, and policy-makers on how to help countries to strengthen their competitive advantage during critical periods marked by extraordinary and globally unfolding events, such as financial crises or pandemics.

More generally, our study contributes to the ongoing debate on how, during more turbulent times, the design of supply chains should take advantage of new opportunities for increasing flexibility and sustainability, and for finding value-creating paths that combine openness and security^51–53^. The recent unfolding of geopolitical disruptions, the COVID-19 pandemic, and various climate-related events have highlighted how vulnerable global value chains can be to exogenous shocks, and how disturbances originating within certain industries or geographic regions may quickly spill over to other parts of the economy^54,55^. As the world starts to reshape supply-chain strategies, the costs of intermediate inputs and factors of production are no longer the only primary determinant of how suppliers are selected. Beyond the standard cost optimization problem, a natural path for countries and industries aiming to secure stability and flexibility of input supplies is to redirect their resources by focusing on domestic alternatives and reducing dependence on offshore connections.

Recent events have clearly illustrated how a country’s excessive reliance on input suppliers that are not geographically close or even politically aligned can heighten the risk of localized disruptions that can, in turn, propagate through the world economy along the various linkages and layers of the global value chains. For example, the ongoing armed conflict in Europe’s breadbasket has triggered a rise in wheat prices and a supply shock that is ripping through the world economy. In addition, many European countries are expected to face gas shortages and a squeeze on nickel used in batteries. Just as occurred after the 2008 financial crisis, it looks increasingly likely that, as the conflict continues, new winners and losers will emerge amid an increasing degree of volatility. Similarly, the conflict will not affect all industries equally. While pricey inputs can severely affect industries further up the value chain (e.g., carmakers, airlines), there are other industries that may benefit from the turmoil, including armsmakers and commodities firms.

Other examples of how exogenous shocks can exacerbate strains on global supply chains that may prove too fragile to depend on include changes in environmental policies and the COVID-19 pandemic. First, environmental policies that adjust the cost of logistics to promote technological decarbonization and reduce global warming may trigger localized micro disturbances potentially reverberating throughout the global value chains^56^. According to the OECD^57^, maritime shipping accounts for around 80% of global trade by volume. Because marine transportation relies primarily on fossil fuels, it is reasonable to expect that policies and tariffs could serve as an incentive for countries to transform their current geographically dispersed production network into a more modular and localized one.

Second, a comparison between the 2008 financial crisis and the current economic downturn induced by the COVID-19 pandemic is inevitable. Like the previous global systemic crisis, COVID-19 is an exogenous shock to the economy, likely to induce structural changes and a repositioning of countries in the global value chains. Indeed, the COVID-19 pandemic has shown that the suppliers’ ability to attend to demand can be at risk if a country or industry cannot cope with the pace of production at the other stages in the global value chains. For instance, the recent “chip shortage” has directly affected the automotive industry^58^ forcing carmakers to implement a shutdown of the production of certain models. In response to that, many countries are now investing in onshore alternatives to avoid further shortages and contain disruptions.

These events are an indication of just how pervasive the knock-on effects of exogenous shocks can be on countries and industries grappling with supply chains. They are also an indication of how countries and industries can realize the full potential of global production networks through a proper redesign of supply chains that transforms supply shocks into opportunities for growth. While there are certainly many ways in which this can be achieved, our discussion of the 2008 financial crisis and of more recent threats to globalization has suggested that a more geographically localized trade network, organized into economic blocs that are politically aligned, may be a response to deal with a global crisis or idiosyncratic shocks to individual countries and industries. All this raises an intriguing possibility: despite the hardships and economic losses countries suffer in the short term, it is possible that shocks to the economy turn out to be strategic inflection points. They can be the start of a sharp decline or the opportunity to rise to new heights. In an increasingly perilous global economy, the 2008 crisis can certainly offer useful insights to policy-makers and governments on how to redesign effective geo-economic strategies to chart countries’ way forward and redefine their global roles. The new emphasis on production reshoring and relocation of supply chains, the current climate crisis, the accelerating technological revolution, and the fast-changing geopolitical landscape will likely set the scene for a new balance of power between countries, and the emergence of new winners and losers in a reshaped world order.

## Methods

### Data

Our study draws on data from the WIOD (Release 2016) covering 28 EU countries and 15 other major countries in the world within the period from 2000 to 2014. For every year, a World Input-Output Table (WIOT) is provided in current prices, expressed in millions of US dollars (USD). Each table represents economic transactions among the 56 economic activities (industries) in each country. The core of the database is a set of harmonized supply and use tables, as well as data on the international trade of goods and services. These two sets of data have been integrated into sets of inter-country WIOT. The full lists of countries and industries are provided in Supplementary Information (Supplementary Tables I, II).

### The multi-layer network

We define our world multi-layer network as a pair $M=(G^{M},C^{M})$, where $G^{M}=\{G^{M}_{\alpha };~\alpha ~\in ~\{1,\dots ,k\}\}$ is a family of directed graphs $G^{M}_{\alpha }=(V^{M}_{\alpha },E^{M}_{\alpha },W^{M}_{\alpha })$ associated with the layers of *M*, and $C^{M}$ is the set of interconnections between nodes belonging to different graphs $G^{M}_{\alpha }$ and $G^{M}_{\beta }$ with $\alpha \ne \beta$. Formally, $C^{M}=\{C^{M}_{\alpha \beta };~\alpha ,~\beta ~\in ~\{1,\dots ,k\}, \alpha \ne \beta \}$ is a family of directed graphs $C^{M}_{\alpha \beta }=\{(i,j)\}$, where $\{i,j\, \in \{1,\dots , N\}\}$, $i \in V^{M}_{\alpha }$ and $j \in V^{M}_{\beta }$. We can further define the element $a_{ij}^{M[\alpha ]}$ of the intra-layer adjacency matrix $A^{M[\alpha ]}$ of each graph $G^{M}_{\alpha }$ as

6 $$\begin{aligned} \begin{array}{c} a_{ij}^{M[\alpha ]} = {\left\{ \begin{array}{ll} w_{ij}^{M[\alpha ]}, &{} \quad \text {if } (i,j) \in E^{M}_{\alpha },\\ 0, &{} \quad \text {otherwise},\\ \end{array}\right. }\\ \end{array} \end{aligned}$$

where $1 \le i,j \le N$; $1 \le \alpha \le k$; $c_i, c_j \in V^{M}_{\alpha }$; and $w_{c_ic_j}^{M[\alpha ]}$ is the sum of the weights associated with all transactions originating from country $c_i$ within a particular industry $\alpha$ and directed to country $c_j$ within the same industry $\alpha$. Thus, an intra-layer edge between country $c_i$ and country $c_j$ in industry $\alpha$ is established when there is at least one transaction between $a_i$ and $a_j$ in $\alpha$.

The element $a_{ij}^{M[\alpha \beta ]}$ of the cross-layer adjacency matrix $A^{M[\alpha \beta ]}$ corresponding to the set of interconnections $C^{M}_{\alpha \beta }$ can be defined as

7 $$\begin{aligned} a_{ij}^{M[\alpha \beta ]} = {\left\{ \begin{array}{ll} w_{ij}^{M[\alpha \beta ]}, &{} \quad \text {if } (i, j) \in C^{M}_{\alpha \beta }, \\ 0, &{} \quad \text {otherwise,}\\ \end{array}\right. } \end{aligned}$$

where $1 \le i, j \le N$; $1 \le \alpha , \beta \le k$; $\alpha \ne \beta$; $c_i \in V^{M}_{\alpha }$; $c_j \in V^{M}_{\beta }$, and $w_{ij}^{M[\alpha \beta ]}$ is the sum of the weights associated with all transactions originating from country $c_i$ within a particular industry $\alpha$ and directed towards country *i* in industry $\beta$. Thus, a cross-layer edge between country *i* in industry $\alpha$ and country *j* in industry $\beta$ is established when there is at least one transaction between $a_i$ and $a_j$ across the corresponding industries.

### Dendrogram clustering

We constructed the dendrograms using hierarchical clustering based on the correlation distance matrix obtained by

8 $$\begin{aligned} d=\sqrt{2 (1-\rho (\theta _i(t),\theta _j(t)))}, \end{aligned}$$

calculated over every pair of countries, where $\theta _i(t)$ is the time series of eigenvector centralities of country *i* and *d* is the distance defined in the interval [0, 2]. We further used the Ward’s linkage criteria to obtain the dendrogram, as implemented in the *Python* package *Scipy*^59^.

To determine the number of clusters, we found the threshold distance that maximizes the silhouette score^44^. This coefficient quantifies the consistency of the clustering procedure and is defined by the average value of

9 $$\begin{aligned} s_i = \frac{b_i-a_i}{\max (a_i,b_i)}\,, \end{aligned}$$

where $a_i$ is the cohesion (the average intra-cluster distance) and $b_i$ is the separation (the average nearest-cluster distance) for the *i*-th country. The higher value of the silhouette coefficient represents the best cluster configuration. We used the Python module *scikit-learn*^60^ to compute the silhouette scores and the *SciPy*^59^ package to compute the correlation distance matrix (Supplementary Fig. 1).

### Null models to assess clusters

To ascertain whether the obtained clusters differ from those obtained with more traditional network partitioning based on the tendency of countries to trade more value within than between blocs, we proceeded as follows. We constructed three null models in which the original simplex trade network maintains its topology (i.e., the in- and out-degree distributions) and is otherwise randomized according to three reshuffling procedures. In the first model (Model I), for each buyer (seller), the weighted stub of each incoming (outgoing) edge is connected with the unweighted stub of an outgoing (incoming) edge chosen uniformly at random across the entire network. In this way, buyers (sellers) preserve their in-strength (out-strength) but are randomly assigned different suppliers (destination markets). In the second model (Model II), weights are reshuffled globally across the network, which thus randomizes nodes’ in- and out-strength. Finally, in the third model (Model III), weights are reshuffled locally across each buyer’s (seller’s) incoming (outgoing) edges. In this way, each node’s in-strength (out-strength) is preserved while its out-strength (in-strength) is randomized. For each model, 1, 000 network realizations were produced and compared to the original network.

### Synthetic multi-layer network (null model)

Given a multi-layer network $M=(G^{M},C^{M})$, we used random edge assignment to construct 1, 000 synthetic multi-layer network realizations that preserve the in- (out-) degree, in- (out-) strength, and weight distributions of the family of observed directed graphs $G^M_\alpha$ (within-layer graphs) and $C^M_{\alpha \beta },\alpha \ne \beta$ (cross-layer graphs). In practice, we randomized the columns (incoming links pointing to buyers) or rows (outgoing links departing from sellers) by blocks of the multi-layer adjacency matrix, where each block includes intra-layer connections (i.e., diagonal matrices given by $G^M_\alpha$), or the inter-layer connections (i.e., off-diagonal matrices given by $C^M_{\alpha \beta },\alpha \ne \beta$).

## Supplementary Information

Supplementary Information.

## Data Availability

The WIOD data is freely available for download at http://www.wiod.org/database/wiots16.

## Code availability

Our code will be available for download at https://github.com/lgaalves/localization after publication.
