# Supplementary material for: The rise and fall of countries in the global value chains
Source: Sci Rep. 2022 May 31;12:9086. doi: 10.1038/s41598-022-12067-x (PMC9154043; doi:10.1038/s41598-022-12067-x)
Supplement: Supplementary file 1 — Supplementary Information. [file 41598_2022_12067_MOESM1_ESM.pdf]

Supplementary Information for
The rise and fall of countries in the global value chains

Luiz G. A. Alves[1], Giuseppe Mangioni[2], Francisco A. Rodrigues[3], Pietro Panzarasa[4,*], and Yamir Moreno[5,6,7]

[1]Department of Chemical and Biological Engineering, Northwestern University, Evanston, IL 60208, USA
[2]Dipartimento di Ingegneria Elettrica, Elettronica e Informatica, University of Catania, Catania 95125, Italy
[3]Institute of Mathematics and Computer Science, University of São Paulo, São Carlos, SP 13566-590, Brazil
[4]School of Business and Management, Queen Mary University of London, London E1 4NS, UK
[5]Institute for Biocomputation and Physics of Complex Systems (BIFI), University of Zaragoza, Zaragoza 50009, Spain
[6]Department of Theoretical Physics, University of Zaragoza, 50009 Zaragoza, Spain
[7]ISI Foundation, Turin 10126, Italy
[*]Correspondence to p.panzarasa@qmul.ac.uk

Data description

Supplementary Table I shows the list of the 43 countries (excluding the "Rest of the World"), and Supplementary Table II the 56 NACE Rev.2 economic activities included in the WIOD.

**Country name (ISO Alpha-3 Code)**
Australia (AUS), Austria (AUT), Belgium (BEL), Bulgaria (BGR), Brazil (BRA), Canada (CAN), Switzerland (CHE), China (CHN), Cyprus (CYP), Czech Republic (CZE), Germany (DEU), Denmark (DNK), Spain (ESP), Estonia (EST), Finland (FIN), France (FRA), United Kingdom (GBR), Greece (GRC), Croatia (HRV), Hungary (HUN), Indonesia (IDN), India (IND), Ireland (IRL), Italy (ITA), Japan (JPN), Korea, Rep. (KOR), Lithuania (LTU), Luxembourg (LUX), Latvia (LVA), Mexico (MEX), Malta (MLT), Netherlands (NLD), Norway (NOR), Poland (POL), Portugal (PRT), Romania (ROU), Russian Federation (RUS), Slovak Republic (SVK), Slovenia (SVN), Sweden (SWE), Turkey (TUR), Taiwan (TWN), United States (USA)

Supplementary Table I. Countries in the WIOD 2016 Release

NACE Rev. 2 Division	Description of economic activities
A01	Crop and animal production, hunting and related service activities
A02	Forestry and logging
A03	Fishing and aquaculture
B	Mining and quarrying
C10-C12	Manufacture of food products, beverages and tobacco products
C13-C15	Manufacture of textiles, wearing apparel and leather products
C16	Manufacture of wood and of products of wood and cork, except furniture; manufacture of articles of straw and plaiting materials
C17	Manufacture of paper and paper products
C18<br>C19	Printing and reproduction of recorded media<br>Manufacture of coke and refined petroleum products
C20	Manufacture of chemicals and chemical products
C21	Manufacture of basic pharmaceutical products and pharmaceutical preparations
C22	Manufacture of rubber and plastic products
C23	Manufacture of other non-metallic mineral products
C24	Manufacture of basic metals
C25	Manufacture of fabricated metal products, except machinery and equipment
C26	Manufacture of computer, electronic and optical products
C27	Manufacture of electrical equipment
C28	Manufacture of machinery and equipment n.e.c.
C29	Manufacture of motor vehicles, trailers and semi-trailers
C30	Manufacture of other transport equipment
C31_C32	Manufacture of furniture; other manufacturing
C33	Repair and installation of machinery and equipment
D35	Electricity, gas, steam and air conditioning supply
E36	Water collection, treatment and supply
E37-E39	Sewerage; waste collection, treatment and disposal activities; materials recovery; remediation activities and other waste management services
F	Construction
G45	Wholesale and retail trade and repair of motor vehicles and motorcycles
G46	Wholesale trade, except of motor vehicles and motorcycles
G47	Retail trade, except of motor vehicles and motorcycles
H49	Land transport and transport via pipelines
H50	Water transport
H51	Air transport
H52	Warehousing and support activities for transportation
H53	Postal and courier activities
I	Accommodation and food service activities
J58	Publishing activities
J59_J60	Motion picture, video and television program production, sound recording and music publishing activities; programming and broadcasting activities
J61	Telecommunications

Continued on next page

Supplementary Table II – continued from previous page

NACE Rev. 2 Division	Description of economic activity
J62_J63	Computer programming, consultancy and related activities; information service activities
K64	Financial service activities, except insurance and pension funding
K65	Insurance, reinsurance and pension funding, except compulsory social security
K66	Activities auxiliary to financial services and insurance activities
L68	Real estate activities
M69_M70	Legal and accounting activities; activities of head offices; management consultancy activities
M71	Architectural and engineering activities; technical testing and analysis
M72	Scientific research and development
M73	Advertising and market research
M74_M75	Other professional, scientific and technical activities; veterinary activities
N	Administrative and support service activities
O84	Public administration and defense; compulsory social security
P85	Education
Q	Human health and social work activities
R_S	Other service activities
T	Activities of households as employers; undifferentiated goods- and services-producing activities of households for own use
U	Activities of extraterritorial organizations and bodies

Supplementary Table II. **Economic activities in WIOD 2016 Release**

Hierarchical structure of the multi-layer network

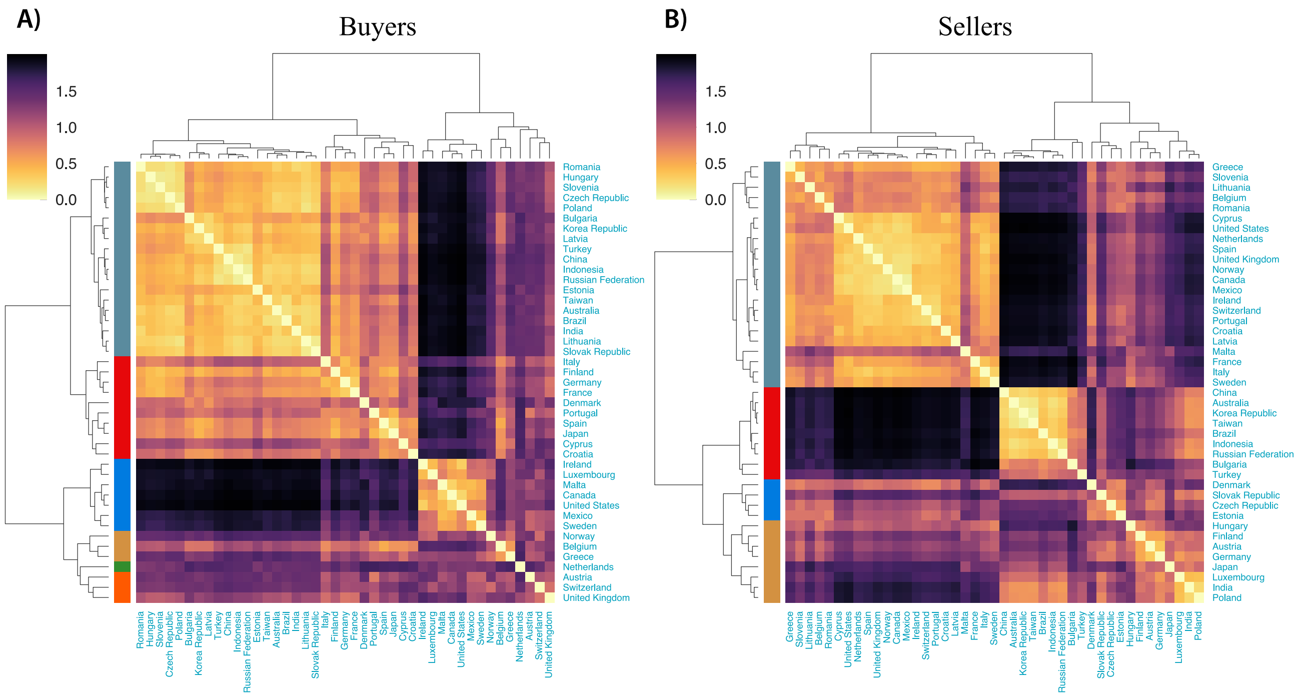

Supplementary Fig. 1. Hierarchical structure of the multi-layer network. Matrix plot of the correlation distance among all pairs of time series of eigenvector centrality for buyers (**A**) and sellers (**B**). The color of each cell is proportional to the correlation distance between the corresponding time series. The dendrograms associated with this matrix show the hierarchical clustering based on Ward's minimum variance method. The colored squares located below the dendrogram branches indicate the clusters obtained by cutting the dendrogram at the threshold distance that maximizes the silhouette score. The buyers' distance matrix has 6 clusters, whereas the sellers' distance matrix only 4 clusters.

Hierarchical (nested) stochastic block model

In addition to the hierarchical clustering analysis of the time series, we considered the hierarchical (nested) stochastic block model (nested SBM) to find the economic blocs[1]. We computed the modular/block structure of the network obtained from the correlation distance matrix of the time series $\theta_i(t)$ of eigenvector centralities. In this network, each node is a country and the weights of links are the correlation distances d_{ij} between the time series of countries i and j. To run the nested SBM algorithm, we considered normal priors for the weight distribution and collected the partitions for $10,000$ sweeps of a Metropolis-Hastings acceptance-rejection Markov Chain Monte Carlo[2] with multiple moves to sample hierarchical network partitions, at intervals of 10 sweeps. The block structure obtained with the hierarchical (nested) SBM for buyers and sellers are shown in Supplementary Figure 2A and Supplementary Figure 2B, respectively. The different colors represent the economic blocs and the adjacency edges are bundled together for a better visualization of the network hierarchical structure.

We further estimated the marginal probabilities of node membership using the fraction of times a node is found in a given partition on our sampled partitions data. In Supplementary Figure 2, the pie charts illustrate the marginal probabilities (fractions of occurrence on the sampled data) that a given node belongs to a partition.

We next compared the results of the nested SBM with the results obtained with the hierarchical clustering analysis of the time series. To do so, we use the normalized mutual information (NMI) to quantify the overlap between the clusters found with the two approaches. Results suggest a great overlap between the results found with the two approaches. The NMI is ≈ 0.7 for the buyers, and ≈ 0.6 for sellers, thus highlighting the robustness of our findings.

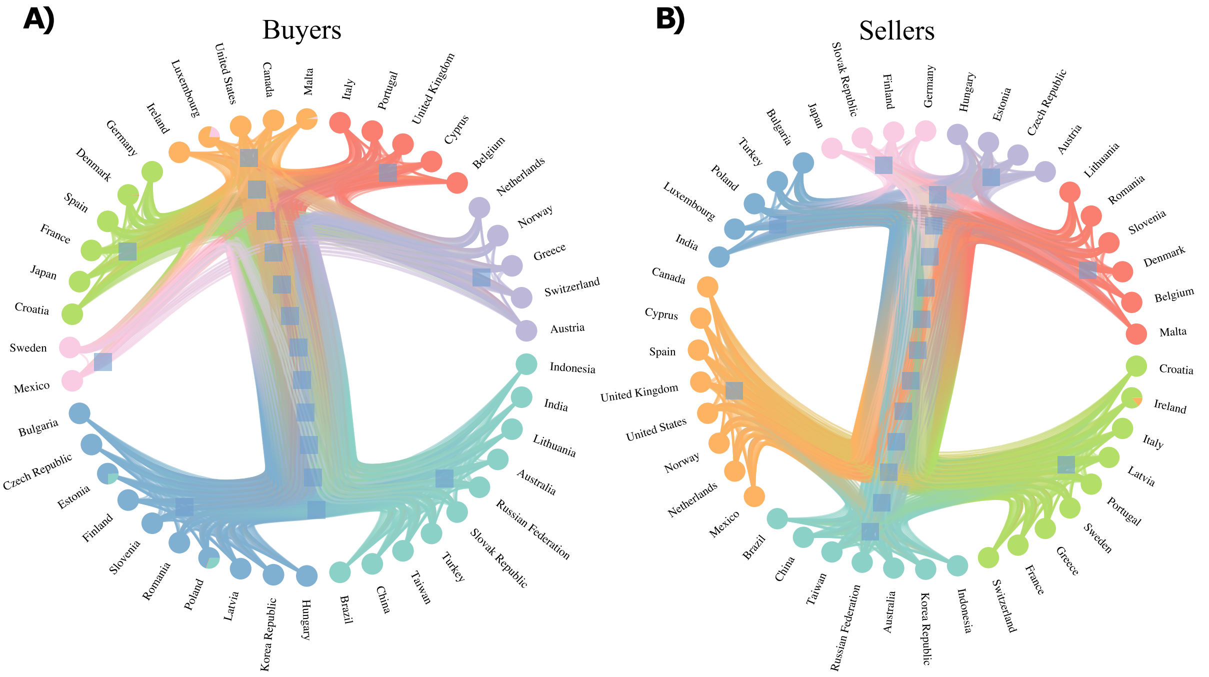

Supplementary Fig. 2. Hierarchical modular structure of the correlation network and marginal probabilities of node membership. In this representation, nodes represent countries, and the pie divisions represent the marginal posterior probability that a node belongs to a given group (the different colors). The probabilities were obtained by collecting the node membership for $10,000$ sweeps of a Metropolis-Hastings acceptance-rejection Markov Chain Monte Carlo with multiple moves to sample hierarchical network partitions, at intervals of 10 sweeps. The edges and their weights are proportional to the correlation distance of the eigenvalue time series $\theta_i(t)$ and $\theta_j(t)$ associated with country i and j. **A**) Modular structure of buyers' correlation network shows a great overlap with the hierarchical cluster analysis (NMI≈ 0.7). **B**) Similarly, the modular structure of sellers' correlation network shows a great overlap with the hierarchical cluster analysis (NMI≈ 0.6).

Fraction of purchases and sales by economic bloc

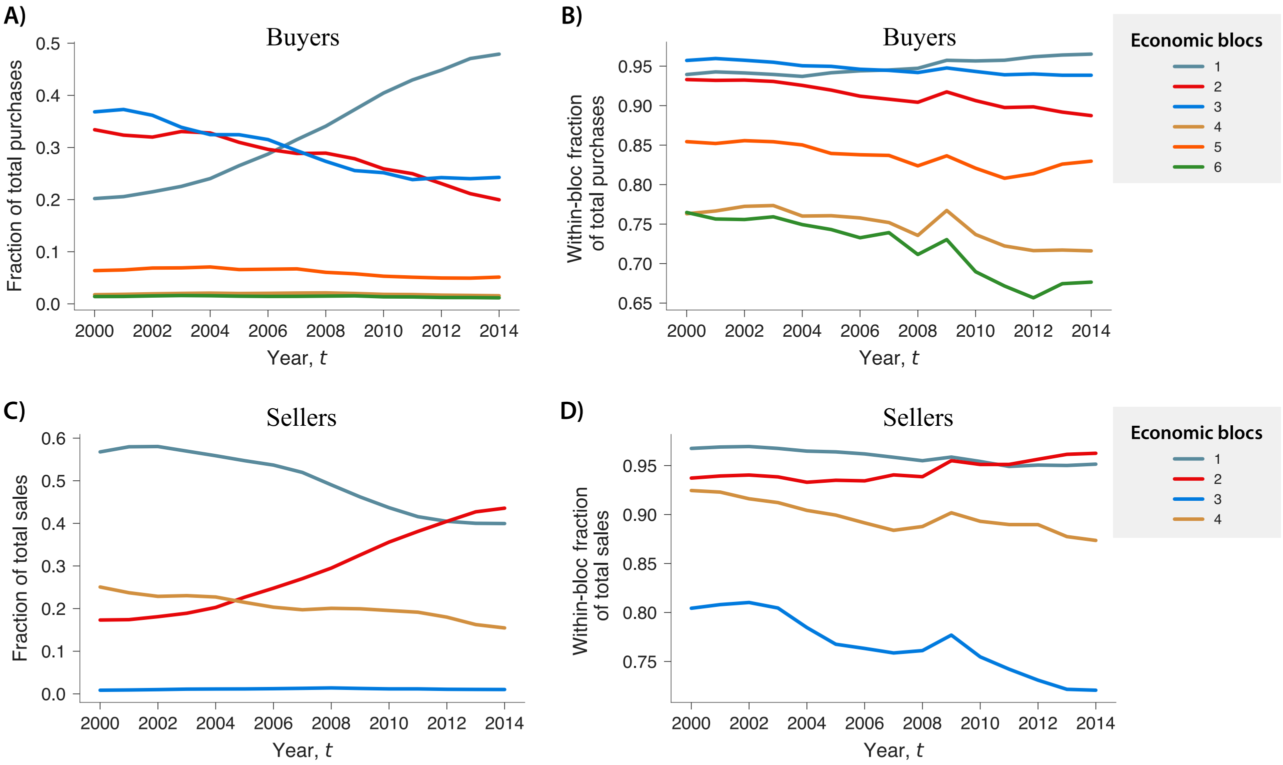

Supplementary Fig. 3. Fraction of purchases and sales by economic bloc. In all plots, the color of each line matches the color of the corresponding economic bloc identified in the hierarchical clustering analysis shown in Supplementary Figure 1. **A**) Fraction of total purchases made by members of each economic bloc as a function of time. The green line represents the largest bloc (in terms of number of countries) which in 2007 secured the largest share of purchases. **B**) Within-bloc fraction of the total purchases made by members of each economic bloc as a function of time. **C**) Fraction of total sales by members of each economic bloc as a function of time. The orange line represents the third largest bloc (in terms of number of countries) which in 2013 secured the largest share of sales. **D**) Within-bloc fraction of the total sales by members of each economic bloc as a function of time. Notice that, for both buyers and sellers, the purchases and sales include domestic and international trade (see Supplementary Figure 4 showing purchases and sales excluding domestic trade).

Fraction of imports and exports by economic bloc

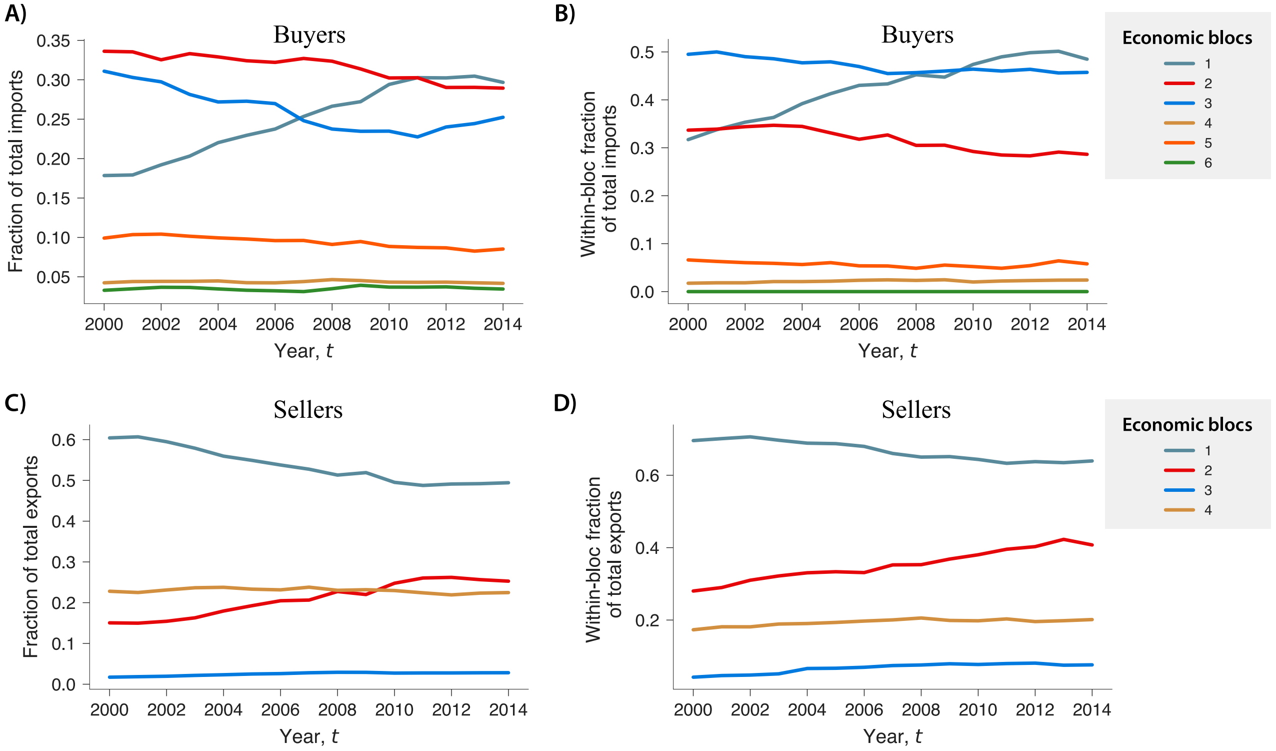

Supplementary Fig. 4. Fraction of imports and exports by economic bloc. In all plots, the color of each line matches the color of the corresponding economic bloc identified in the hierarchical clustering analysis shown in Supplementary Figure 1. **A**) Fraction of total imports by members of each economic bloc as a function of time. The green line represents the third largest bloc (in terms of number of countries) which in 2010 secured the largest share of imports. **B**) Within-bloc fraction of the total imports by members of each economic bloc as a function of time. **C**) Fraction of total exports by members of each economic bloc as a function of time. The orange line represents the third largest bloc (in terms of number of countries) which in 2009 secured the second largest share of sales. **D**) Within-bloc fraction of the total sales by members of each economic bloc as a function of time.

Fraction of domestic purchases and sales by economic bloc

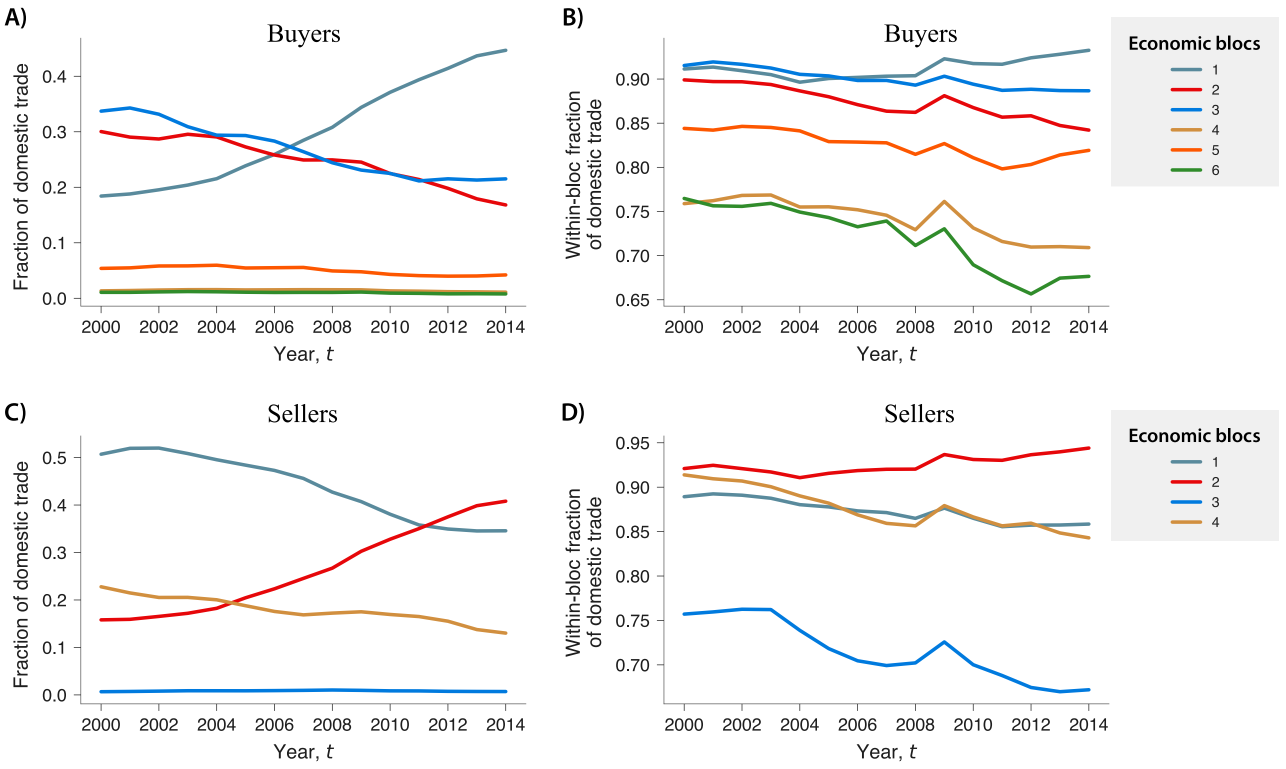

Supplementary Fig. 5. Fraction of domestic purchases and sales by economic bloc. In all plots, the color of each line matches the color of the corresponding economic bloc identified in the hierarchical clustering analysis shown in Supplementary Figure 1. **A**) Fraction of domestic purchases made by members of each buyer economic bloc as a function of time. The green line represents the third largest bloc (in terms of number of countries) which in 2010 secured the largest share of domestic trade. **B**) Within-bloc fraction of the domestic purchases made by members of each economic bloc as a function of time. **C**) Fraction of domestic sales by members of each economic bloc as a function of time. The orange line represents the third largest bloc (in terms of number of countries) which in 2009 secured the second largest share of sales. **D**) Within-bloc fraction of domestic sales by members of each economic bloc as a function of time.

Null models to assess network clustering

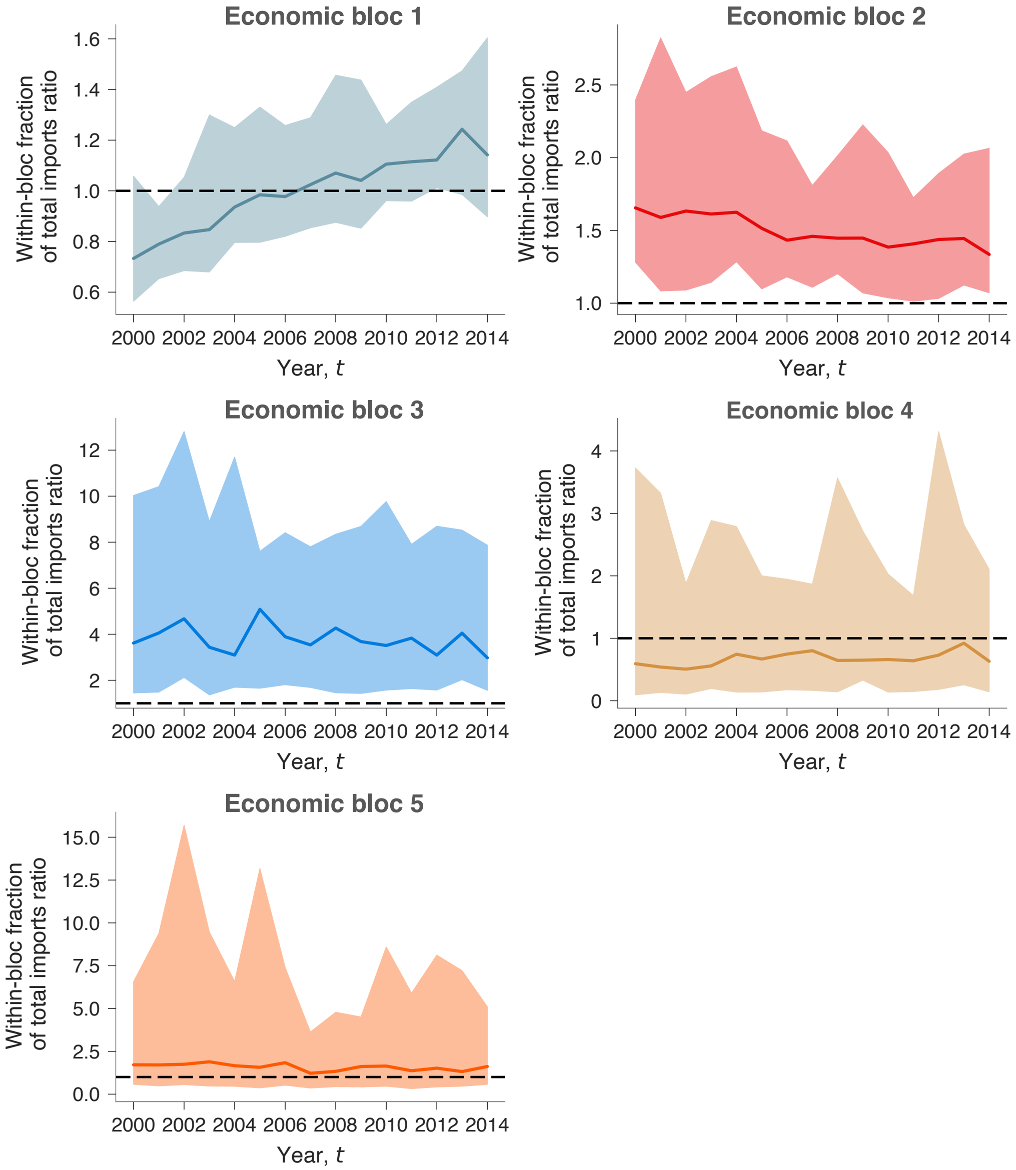

Supplementary Fig. 6. Null models to assess network clustering: Model I (importers). Within-bloc fraction of the total imports by members of each economic bloc as a function of time. In all plots, the color of each line matches the color of the corresponding economic bloc identified in the hierarchical clustering analysis shown in Supplementary Figure 1. The shaded areas represent the 95% confidence intervals obtained from 1,000 realizations of the null model. We omitted the economic bloc no. 6 since it only includes one country.

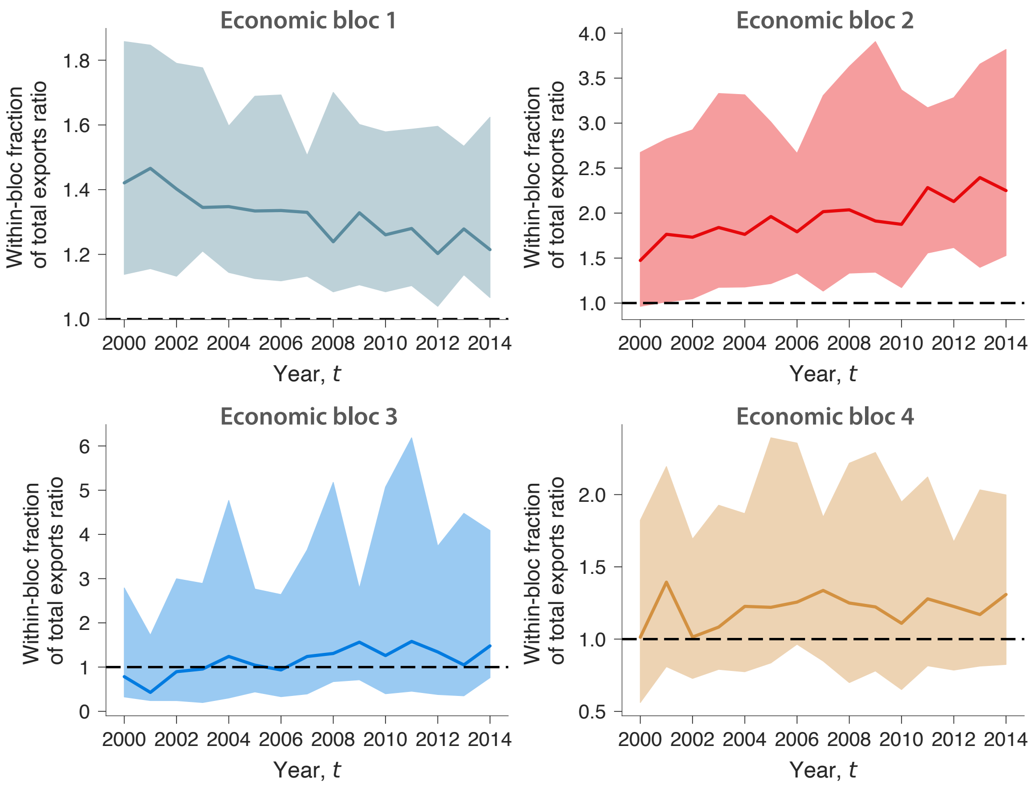

Supplementary Fig. 7. Null models to assess network clustering: Model I (exporters). Within-bloc fraction of the total exports by members of each economic bloc as a function of time. In all plots, the color of each line matches the color of the corresponding economic bloc identified in the hierarchical clustering analysis shown in Supplementary Figure 1. The shaded areas represent the 95% confidence intervals obtained from $1,000$ realizations of the null model.

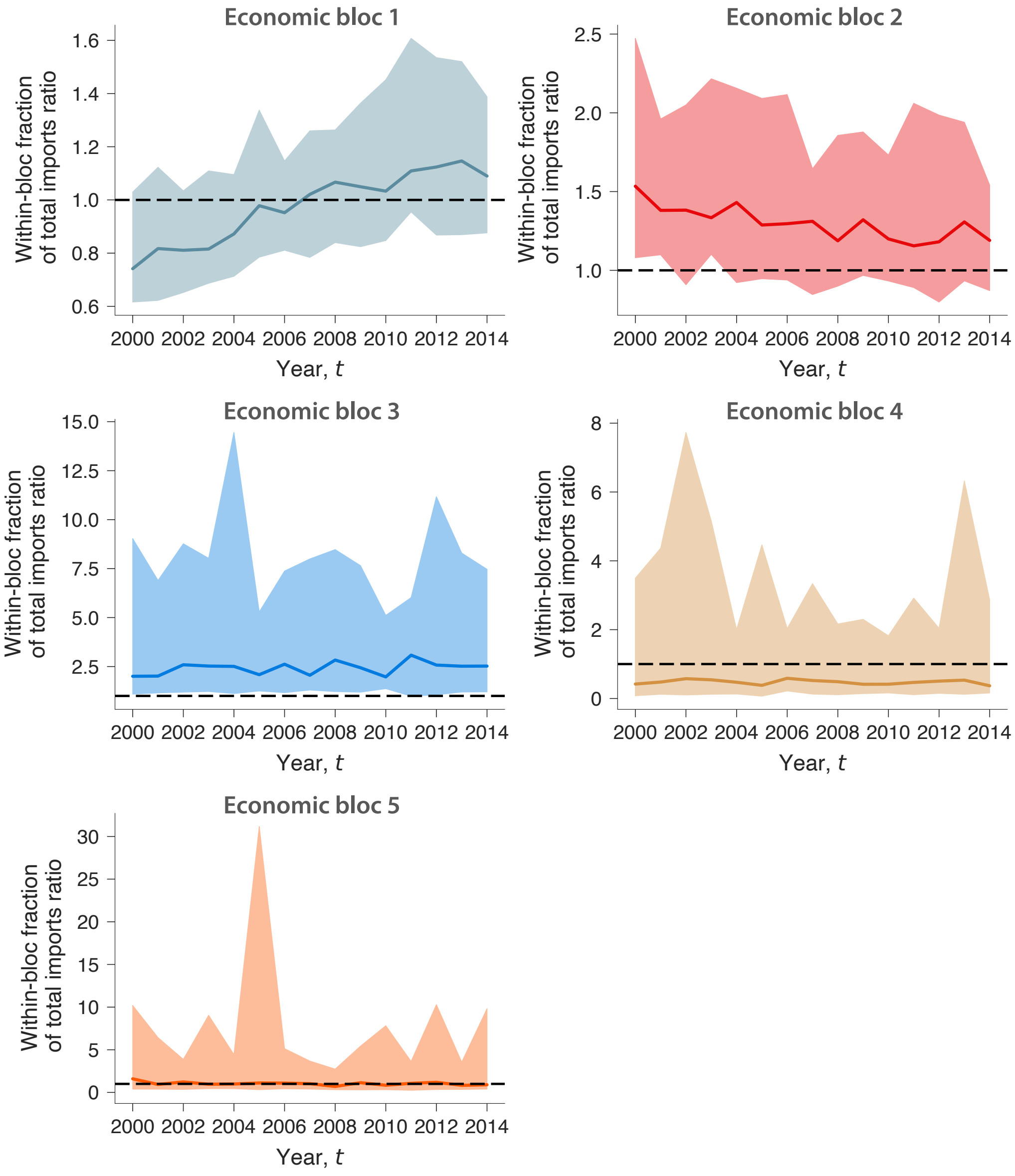

Supplementary Fig. 8. Null models to assess network clustering: Model II (importers). Within-bloc fraction of the total imports by members of each economic bloc as a function of time. In all plots, the color of each line matches the color of the corresponding economic bloc identified in the hierarchical clustering analysis shown in Supplementary Figure 1. The shaded areas represent the 95% confidence intervals obtained from 1,000 realizations of the null model. We omitted the economic bloc no. 6 since it only includes one country.

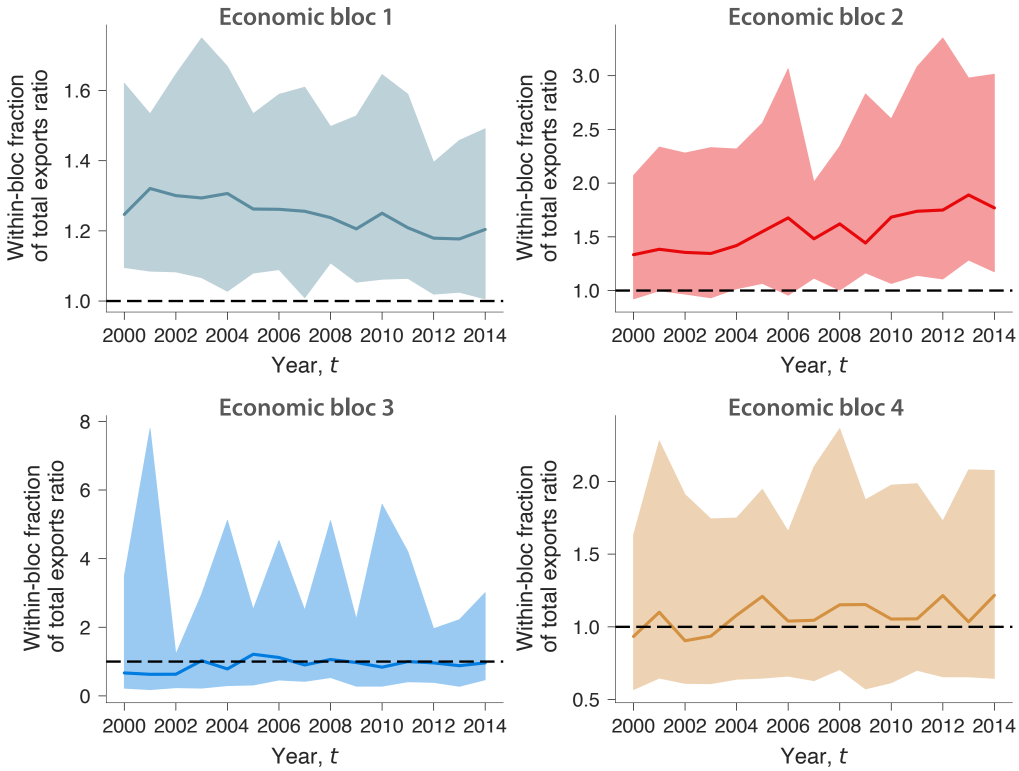

Supplementary Fig. 9. Null models to assess network clustering: Model II (exporters). Within-bloc fraction of the total exports by members of each economic bloc as a function of time. In all plots, the color of each line matches the color of the corresponding economic bloc identified in the hierarchical clustering analysis shown in Supplementary Figure 1. The shaded areas represent the 95% confidence intervals obtained from 1,000 realizations of the null model.

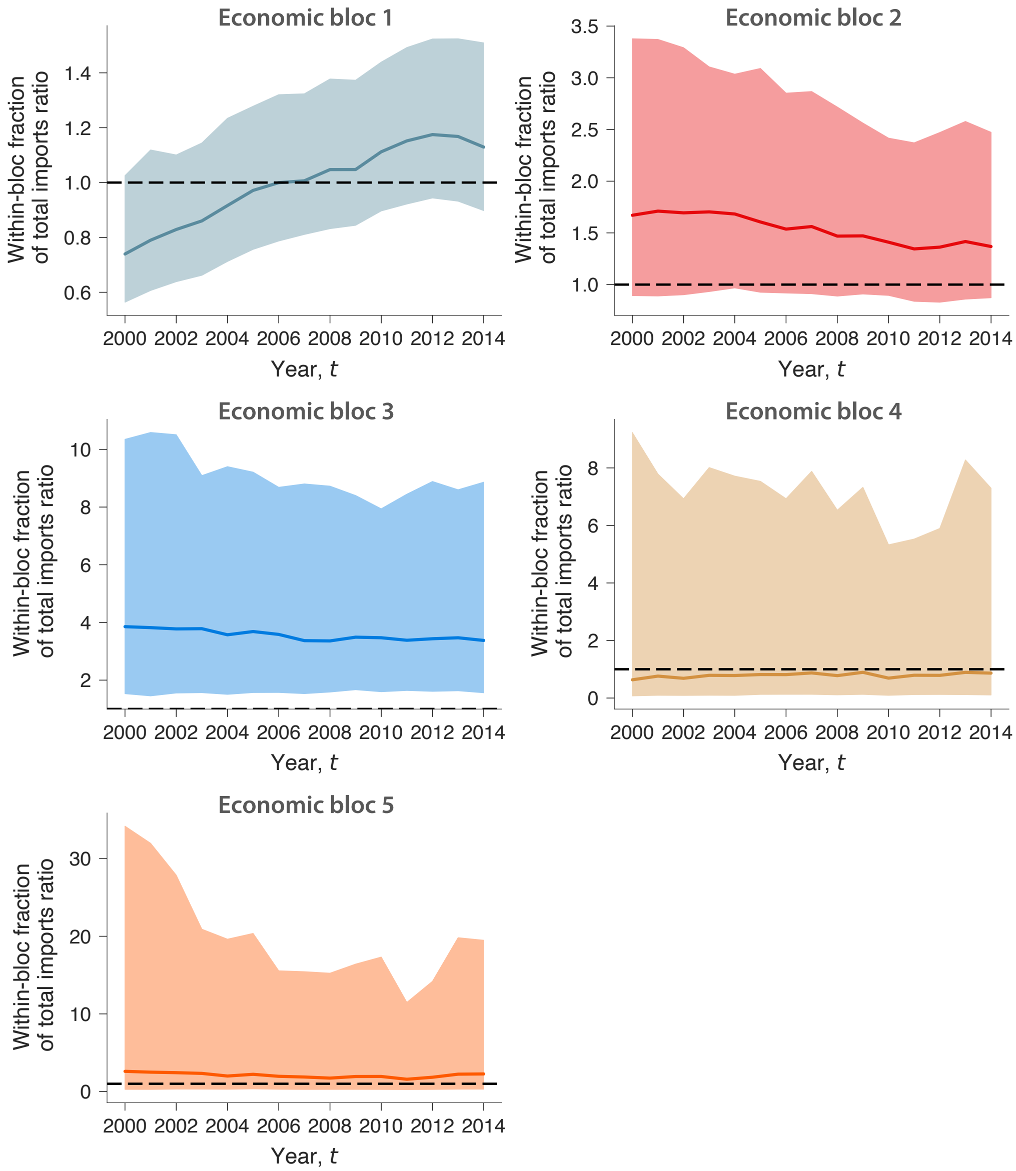

Supplementary Fig. 10. **Null models to assess network clustering: Model III (importers)**. Within-bloc fraction of the total imports by members of each economic bloc as a function of time. In all plots, the color of each line matches the color of the corresponding economic bloc identified in the hierarchical clustering analysis shown in Supplementary Figure 1. The shaded areas represent the 95% confidence intervals obtained from 1,000 realizations of the null model. We omitted the economic bloc no. 6 since it only includes one country.

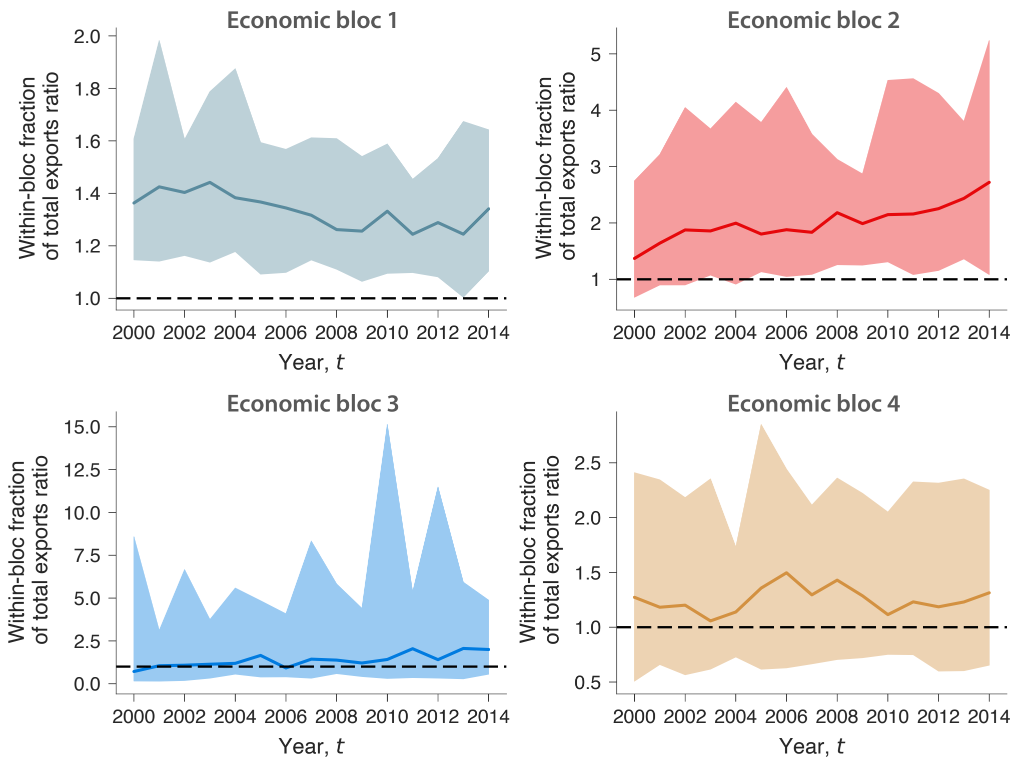

Supplementary Fig. 11. Null models to assess network clustering: Model III (exporters). Within-bloc fraction of the total exports by members of each economic bloc as a function of time. In all plots, the color of each line matches the color of the corresponding economic bloc identified in the hierarchical clustering analysis shown in Supplementary Figure 1. The shaded areas represent the 95% confidence intervals obtained from $1,000$ realizations of the null model.

Geographical mapping of economic blocs

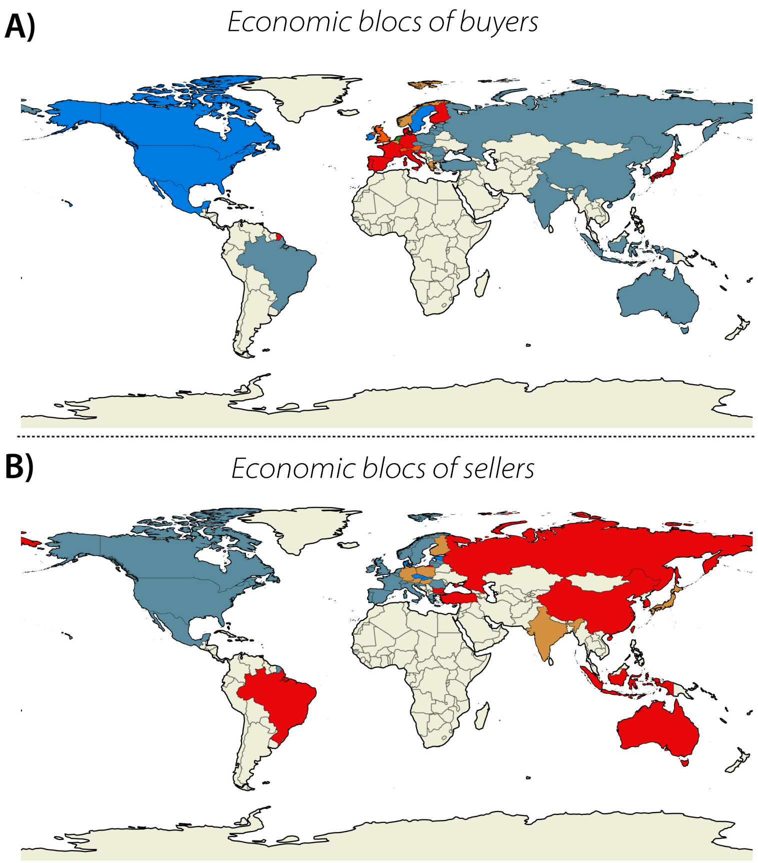

Supplementary Fig. 12. Geographical mapping of economic blocs. Colors of countries match the colors of the corresponding economic blocs identified in the hierarchical clustering analysis shown in Supplementary Figure 1. **A**) Geographical mapping of economic blocs of buyers. **B**) Geographical mapping of economic blocs of sellers. Maps were generated using the Python package *cartopy* version 0.18.0[3].

Mean geodesic distance within and between blocs

Economic bloc	Within-bloc mean distance	Between-bloc mean distance
1	5630.9	5512.42
2	2994.43	4452.62
3	4676.4	5914.79
4	1234.5	3661.09
5	723.47	3589.81
6	0	3397.31

Supplementary Table III. Mean geodesic distance between buyers. The first column indicates the economic blocs of buyers. The second column shows the mean geodesic distance (computed using the geographical centroid of countries) between countries within the same economic bloc. The third column shows the mean distance between each country within a given bloc and all the other countries not in the bloc. Notice that since bloc no. 6 includes only one country, the mean distance is 0. Except for the largest cluster (i.e., economic bloc no. 1), for all the other clusters of buyers within-bloc distances are smaller than between-bloc distances.

Economic bloc	Within-bloc mean distance	Between-bloc mean distance
1	3129.63	5422.89
2	7187.36	7875.84
3	690.5	3523.01
4	3489.43	4532.31

Supplementary Table IV. Mean geodesic distance between sellers. The first column indicates the economic bloc of sellers. The second column shows the mean geodesic distance (computed using the geographical centroid of countries) between countries within the same economic bloc. The third column shows the mean distance between each country within a given bloc and all the other countries not in the bloc. For all clusters of sellers, within-bloc distances are smaller than between-bloc distances.

IPR and comparison with synthetic multi-layer networks

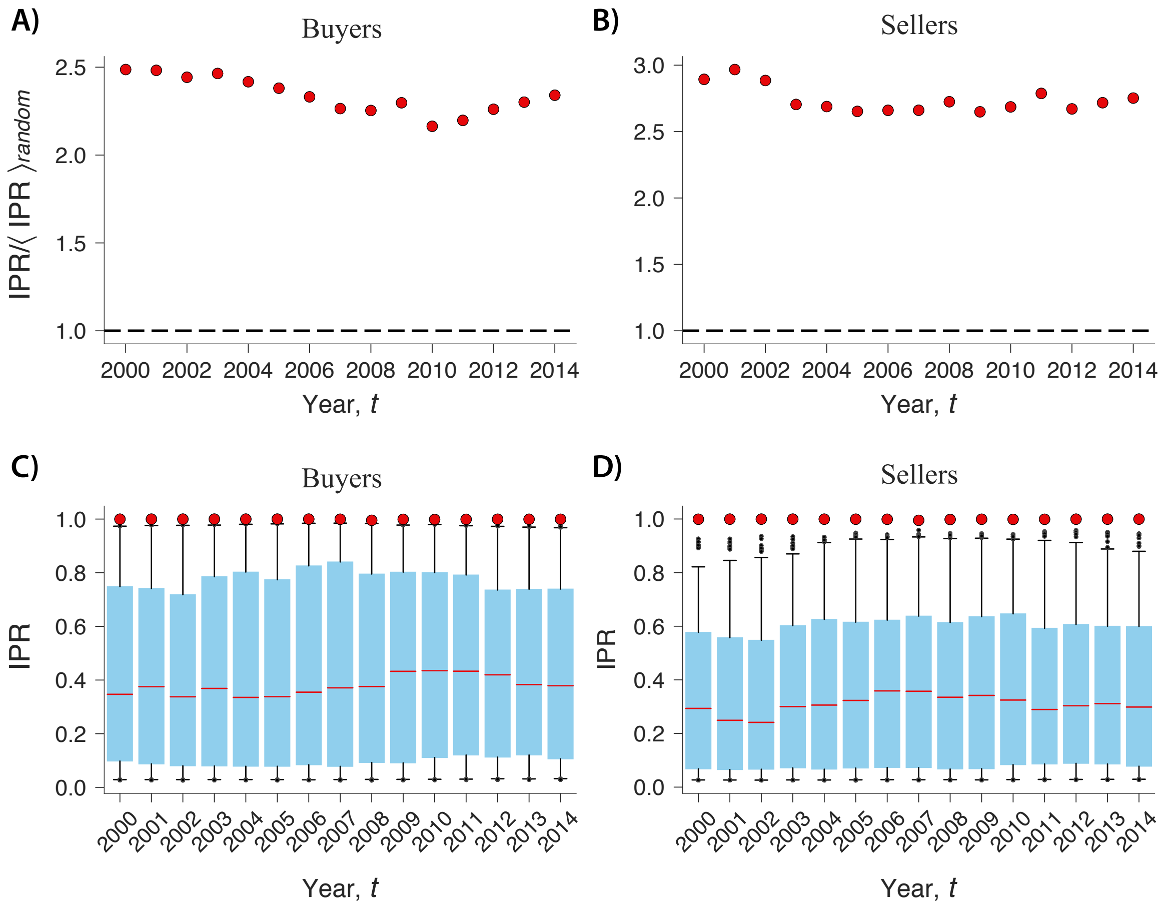

Supplementary Fig. 13. IPR computed on real multi-layer networks compared with the IPR obtained from synthetic random multi-layer network realizations. **A**) Ratio $\mathrm{IPR}/\langle\mathrm{IPR}\rangle_{\mathrm{random}}$ for buyers (red circles). **B**) Ratio $\mathrm{IPR}/\langle\mathrm{IPR}\rangle_{\mathrm{random}}$ for sellers (red circles). If localization observed in the real network can be replicated using random synthetic multi-layer networks, the ratio is close to one (black dashed lines). Panels **A** and **B** suggest that the values of IPR, respectively for buyers and sellers, are almost three times as large as the average values found on the ensembles of random synthetic multi-layer networks. Panels **C** and **D** report the confidence intervals for $\mathrm{IPR}_{\mathrm{random}}$, based on $1,000$ realizations, respectively for buyers and sellers. All values of the observed IPR are statistically significantly different from $\mathrm{IPR}_{\mathrm{random}}$ at the 5% significance level. The box-plot panels show the values from the ensembles of random network realizations for buyers (**C**) and sellers (**D**), and also include the values of the IPR observed in the real networks (red circles). Each blue box represents the two innermost quartiles. The whiskers represent the 95% confidence intervals, the red horizontal lines are the medians, and the small black dots are outliers of the ensembles of synthetic random multi-layer networks.

Critical value for localization transition

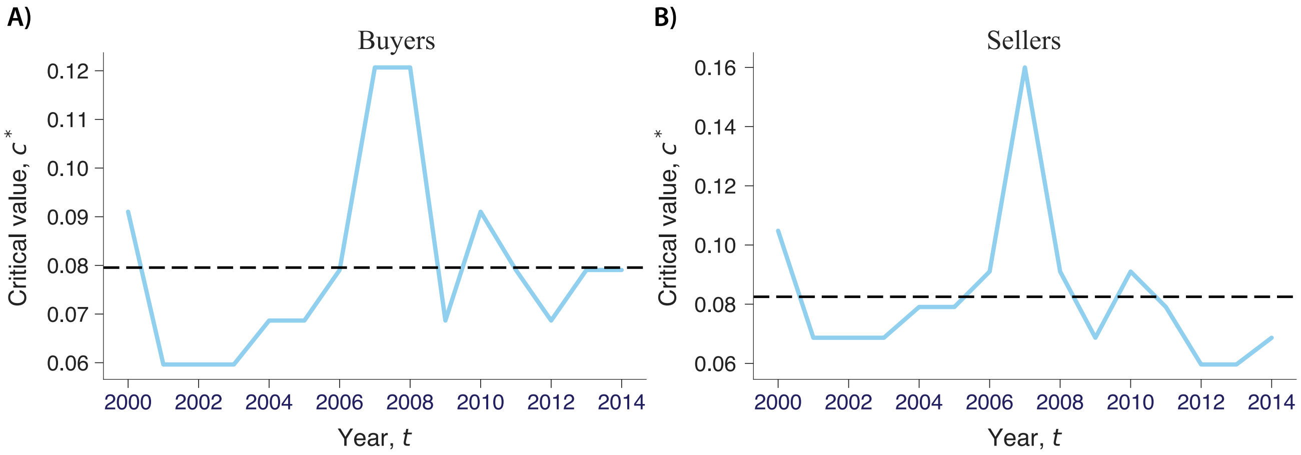

Supplementary Fig. 14. Critical value for localization transition. The critical value is defined as the $c = c^*$ where the largest variation of IPR occurs. The blue lines show the variation of c^* over time and the black dashed lines show the average value over the period, $\langle c^* \rangle \approx 0.08$. **A**) Critical values for buyers reach their maximum in 2007 and 2008. **B**) Critical values for sellers reach their maximum in 2007.

Evolution of international and domestic trade

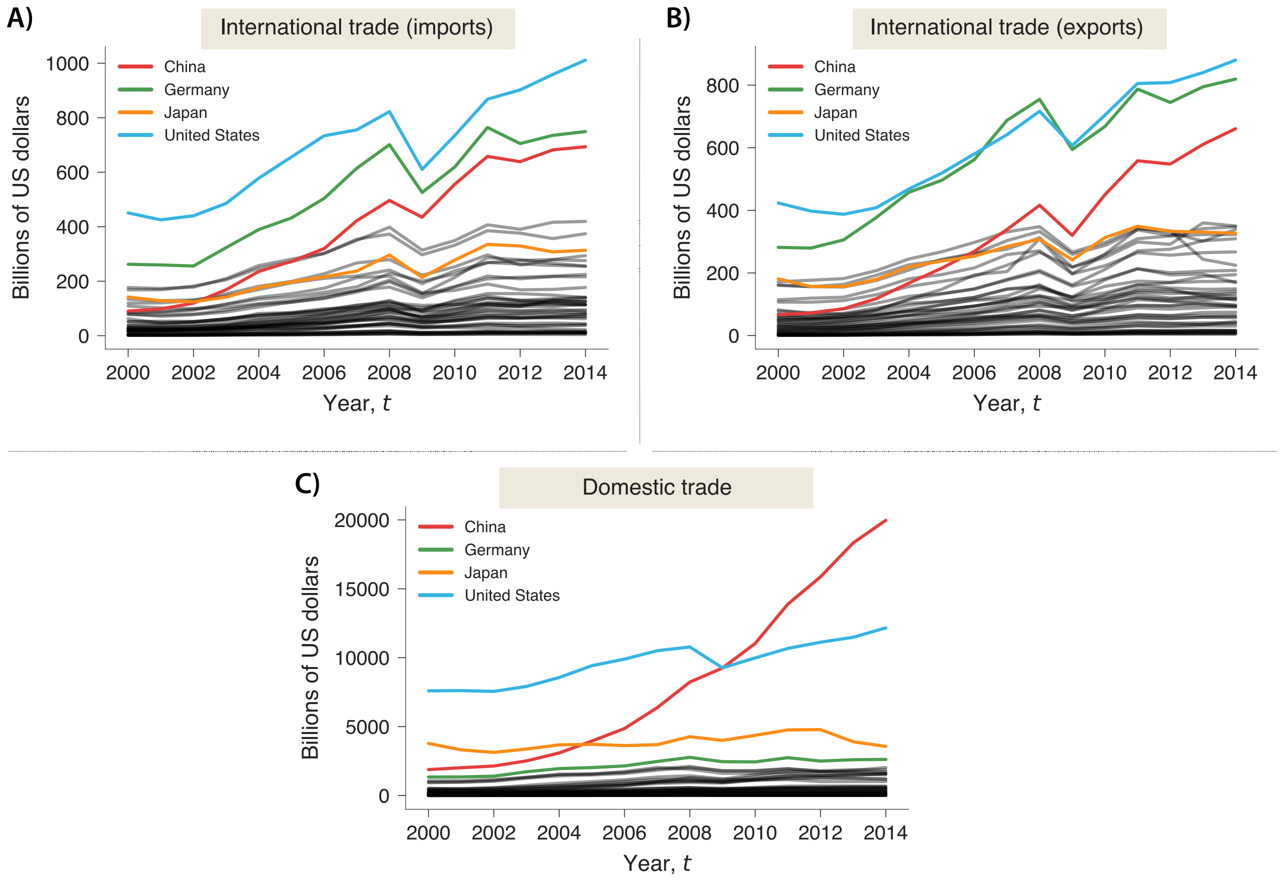

Supplementary Fig. 15. Evolution of countries' international trade and domestic trade. The US has always secured the largest share of imports (**A**) and exports (**B**), while Germany ranked second, and in 2007 China overtook other countries reaching the third position. While Japan was characterized by a significant share of global domestic trade, its share of international trade did not rank as high as the other major countries' share. **C**) Domestic trade significantly increased in China during the observation period, whereas countries such the US, Germany, and Japan witnessed a much smaller growth.

References

1. Peixoto, T. P. Hierarchical block structures and high-resolution model selection in large networks. *Phys. Rev. X* **4**, 011047 (2014).

2. Peixoto, T. P. Efficient Monte Carlo and greedy heuristic for the inference of stochastic block models. *Phys. Rev. E* **89**, 012804 (2014).

3. Met Office. *Cartopy: a cartographic python library with a matplotlib interface*. Exeter, Devon (2010 - 2015). URL http://scitools.org.uk/cartopy.
